# Unraveling the Anti‐Arthritic Potential of Bioactive Compounds From *Melaleuca alternifolia* Oil: Integrated In Vivo, Molecular Docking, and Network Pharmacology Insights

**DOI:** 10.1002/fsn3.72328

**Published:** 2026-09-23

**Authors:** Sana Sabir, Aisha Mobashar, Hossam Kamli, Hafiza Mahnoor Baghdadi, Bushra Akhtar, Amir Ali, Sana Saleem, Khalid Hussain, Kiran Mashaal, Esmael M. Alyami, Mona Alsolami, Fakhreldeen Dabiellil, Pravin Badhe, Gehan M. Elossaily, Muhammad Shahab, Abdel‐Rhman Z. Gaafar

**Affiliations:** ^1^ Faculty of Pharmacy The University of Lahore Lahore Pakistan; ^2^ Faculty of Health Sciences Equator University of Science and Technology Maska Uganda; ^3^ Department of Clinical Laboratory Sciences, College of Applied Medical Sciences King Khalid University Riyadh Saudi Arabia; ^4^ Department of Pharmacy, Faculty of Health and Pharmaceutical Sciences University of Agriculture Faisalabad Pakistan; ^5^ Department of Biology, College of Science King Khalid University Abha Asir Saudi Arabia; ^6^ Research Centre for Advanced Materials Science (RCAMS) King Khalid University Abha Saudi Arabia; ^7^ University of Bahr el Ghazal Wau South Sudan; ^8^ Swalife Biotech LTD IE Sinhgad College of Pharmacy Cork Ireland; ^9^ Department of Basic Medical Sciences College of Medicine, AlMaarefa University Riyadh Saudi Arabia; ^10^ Research Center, Deanship of Scientific Research and Post‐Graduate Studies AlMaarefa University Riyadh Saudi Arabia; ^11^ State Key Laboratories of Chemical Resources Engineering Beijing University of Chemical Technology Beijing China; ^12^ Department of Botany and Microbiology, College of Science King Saud University Riyadh Saudi Arabia

**Keywords:** cytokines, *Melaleuca alternifolia* oil, pro‐inflammatory biomarkers, rheumatoid arthritis

## Abstract

Rheumatoid arthritis (RA) is a chronic inflammatory condition that affects millions of people globally, often leading to morbidity. Recent research highlights the potential of natural products in managing arthritis symptoms and modifying disease progression. Among these, *Melaleuca alternifolia* oil (MAO) has emerged as a promising therapeutic option due to its anti‐inflammatory and analgesic properties. A total of 36 Sprague–Dawley rats weighing between 200 and 220 g were divided into six groups (*n* = 6). On day 0, arthritis was induced by CFA. On day 8, treatment started and lasted till day 28. Paw edema and arthritic score were measured. Moreover, hematological, histological, and biochemical parameters were evaluated. The mRNA expression levels of anti‐inflammatory cytokine IL‐4 and pro‐inflammatory cytokines such as TNF‐α, IL‐6, NF‐κB, and IL‐1β were measured using real‐time polymerase chain reaction (qPCR). ELISA was used to detect PGE2 levels. Network pharmacology was used to evaluate the hub genes between the phytocompounds and rheumatoid arthritis. Molecular docking and scoring calculations were done for the top matched hub genes for moderate confirmations between phytocompounds and targets. GC–MS analysis was also performed to identify potential anti‐inflammatory constituents present in the plant oil. Treatment with MAO effectively reduced paw edema and swelling. Histopathological examinations demonstrated significant improvement in the treatment groups. Biochemical parameters such as creatinine, urea, alanine aminotransferase (ALT), and alkaline phosphatase (ALP) did not significantly alter, and hematological parameters such as red blood cells (RBC), white blood cells (WBC), hemoglobin (Hb), and platelet count were restored in the treated group. The mRNA expression of anti‐inflammatory cytokines, including IL‐4, was upregulated, and pro‐inflammatory biomarkers such as TNF‐α, IL‐1β, IL‐6, and NF‐κB were significantly downregulated in the arthritic control group. Furthermore, treatment with MAO significantly reduced PGE2 levels. Network pharmacology indicated the hub genes and several biological processes, molecular functions, cellular components, and KEGG pathways that are involved in rheumatoid arthritis. Molecular docking was performed against hub genes such as AKT1, BCL‐2, MAPK3, and MMP9 that are involved in various pro‐inflammatory cytokine formation and pathophysiology of rheumatoid arthritis. GC–MS analysis of the plant's oil revealed the presence of numerous anti‐inflammatory and antirheumatic phytochemicals. MAO possesses considerable anti‐arthritic properties by downregulating pro‐inflammatory biomarkers such as IL‐1β, NF‐κB, IL‐6, TNF‐α, and upregulating anti‐inflammatory IL‐4. Further investigations are needed to explore its potential as a natural therapeutic option for treating RA.

## Graphical abstract

*Melaleuca alternifolia* oil demonstrated significant anti‐arthritic effects in CFA‐induced rats by reducing paw edema, PGE2 levels, and inflammatory biomarkers. It improved hematological and histopathological parameters, modulated cytokine expression, and showed promising phytochemical‐target interactions through GC–MS, network pharmacology, and molecular docking.

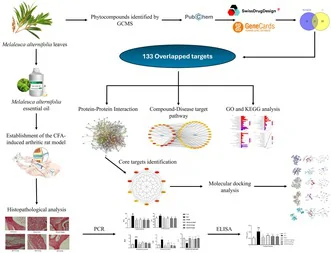

## 1 Introduction

Rheumatoid arthritis (RA) is an autoimmune disorder that affects joints, particularly the hands, wrists, and knees. Bone erosion, cartilage degradation, and joint inflammation are the hallmarks of RA, which leads to pain, stiffness, swelling, fatigue, and weight loss. The pathophysiology of RA is driven by cytokines, including TNF‐α and IL‐6, which stimulate inflammatory responses. TNF‐α induces IL‐6 production, which subsequently activates NF‐κB and IL‐1β cytokines, leading to the production of PGE2, causing further joint damage, discomfort, and pain (Ali et al. 2024; Nazir et al. 2024). Arthritis affects millions worldwide, with osteoarthritis impacting around 595 million people in 2020 and expected to reach 1 billion by 2050. In the U.S, nearly 19% of adults have arthritis, with higher rates in women and older age groups (Meade et al. 2024).

In rheumatoid arthritis, certain inflammatory signals like TNF‐α, IL‐6, IL‐1β, and NF‐κB contribute to swelling, pain, and joint damage. Although the body produces anti‐inflammatory cytokines like IL‐4 to counteract this process, their effect is often not strong enough. This imbalance allows inflammation to persist, leading to progressive damage to joints and surrounding tissues (Mehmood et al. 2025; Zulfiqar et al. 2025).

Non‐steroidal anti‐inflammatory drugs (NSAIDs) and disease‐modifying anti‐rheumatic drugs (DMARDs) often come with significant side effects, including gastrointestinal complications, infection risk, and tolerance issues with opioid use. Corticosteroids are effective for inflammation but can lead to weight gain, high blood sugar, weak bones, mood swings, and a weakened immune system (Chaudhuri et al. 2024; Lisiecka 2024). Similarly, biologic drugs used for autoimmune conditions may increase the risk of infections and certain cancers. Both treatments require careful monitoring to balance their benefits with potential risks. Long‐term use should always be guided by a healthcare professional to minimize complications (Virtanen et al. 2024). NSAIDs are useful for easing pain and swelling but do not stop the disease from getting worse and can cause issues like stomach irritation or kidney strain if used long term. DMARDs help slow the disease but take weeks to show results and may lead to side effects such as infections or liver trouble. Because of these challenges, there is a growing need for treatments that are both safer and more effective (Arfeen et al. 2024; Sen et al. 2024).

New therapeutic approaches, particularly involving plant‐based phytochemicals, have gained interest due to their natural anti‐inflammatory properties (Malik et al. 2025; Nazir et al. 2024). Plant oils are being explored for rheumatoid arthritis because they may have fewer side effects than standard drugs. In recent decades, alternative medicines, including *Melaleuca alternifolia* L. oil (MAO), have gained significant popularity. MAO is one such option, known for its anti‐inflammatory and antioxidant effects. Its key compounds, like terpinen‐4‐ol, may help reduce joint inflammation and protect tissues from damage, making it a potential supportive therapy in RA. This essential oil, which has been utilized in Australia for nearly a century, is now accessible globally in both its pure form and as an ingredient in various products. Traditionally, the primary applications of MAO have leveraged its anti‐inflammatory and antiseptic properties (Sial et al. 2024).

MAO contains terpene hydrocarbons, primarily monoterpenes, sesquiterpenes, and alcohols. MAO contains various bioactive compounds, including terpinen‐4‐ol (40.1%), monoterpene γ‐terpinene (23%), α‐Terpinene (10.4%). 1,8‐cineole (5.1%), ρ‐Cymene (2.9%), Limonene (1%), which have demonstrated considerable anti‐inflammatory effects (Balahbib et al. 2021; Carson et al. 2006; Hoch et al. 2023; Kathem et al. 2024). It has been shown to inhibit the synthesis of pro‐inflammatory cytokines in human monocytes treated with lipopolysaccharide (LPS) as well as in murine RAW264.7 macrophages. The purpose of this work is to explore the network pharmacology, in silico studies, and immunomodulatory role of MAO in CFA‐induced arthritic rats.

## 2 Materials and Methods

### 2.1 Sampling

*Melaleuca alternifolia* L. oil (TTO) was procured from Jiangxi Global Natural Spice Co. Ltd. (Batch No. 20210317‐TTO) (Figure S1). The oil was stored in a dark, airtight container at 4°C until further use. Thirty‐six healthy female Sprague–Dawley rats (200–220 g) were taken from the animal house of The University of Lahore, Lahore, 54000, Pakistan, for this study. The animals were kept in controlled environments with a 12‐h light/dark cycle, temperature: 28°C ± 2°C, humidity: 60%–70% at the University of Lahore. This study received ethical approval from the University of Lahore Research Ethics Committee (IREC‐2017‐23).

### 2.2 Experimental Design and Arthritis Induction

The rats were divided into six groups (*n* = 6). On Day 0, arthritis was induced by a single subplantar injection of 0.15 mL Complete Freund's Adjuvant (CFA) Sigma‐Aldrich (St. Louis, MO, USA) containing heat‐killed *Mycobacterium tuberculosis* (1 mg/mL) into the left hind paw of rats. The successful induction of arthritis was confirmed by the presence of visible paw swelling and elevated arthritic scores in CFA‐treated rats on Day 7 before the commencement of treatment. On Day 8, treatment was initiated and continued until Day 28 (Mobashar et al. 2022). Group I was regarded as the normal control group receiving 1% Tween 80 only, while Group II was considered as diseased control receiving 0.15 mL CFA only. Group III received piroxicam Pfizer Inc., New York, NY, USA (10 mg/kg) as the standard drug control, while groups IV, V, and VI received low‐dose (0.15 mL/kg), medium dose (0.30 mL/kg), and high‐dose (0.60 mL/kg) of MAO, respectively. MAO was given in mL/kg, as is common for essential oils, which is almost equal to 134, 267, and 534 mg/kg, making the doses easy to reproduce while accounting for the oil's density and volatility. Previous studies have shown that oral doses of MAO up to 1 mL/kg are safe and beneficial in rats, particularly in infection and sepsis models. To stay within a safe margin, we selected lower doses of 0.15, 0.30, and 0.60 mL/kg for our anti‐inflammatory study. *M. alternifolia* oil was administered orally after emulsification in 1% Tween 80 to improve its dispersion and facilitate dosing. The treatment groups received 0.15, 0.30, and 0.60 mL/kg of the emulsified oil preparation once daily throughout the treatment period. These doses fall well below the reported oral LD_50_ range of 1.9–2.6 mL/kg (Baldissera et al. 2014; Dar et al. 2024).

### 2.3 Evaluation of Arthritic Score

The arthritic progression was assessed by observing symptoms such as swelling, erythema, and redness, with a scoring system ranging from 0 to 4, where 0 indicated no swelling, 1 indicated mild swelling, 2 indicated moderate swelling, 3 indicated severe swelling, and 4 indicated deformity and inability to use limbs (Mashaal et al. 2023).

### 2.4 Measurement of Paw Volume

Paw volume was measured on 8, 15, 22, and 28, respectively, using a plethysmometer (Abid et al. 2022).

### 2.5 Hematological Parameters

Through heart puncture, blood samples were obtained in Vacutainer containers containing EDTA. Hematological features, such as hemoglobin (Hb), red blood cell (RBC) count, white blood cell (WBC) count, and platelet count, were then determined using an automated hematology analyzer (Dar et al. 2023).

### 2.6 Biochemical Parameters

After being anesthetized with Ketamine/Xylazine (100 mg/kg; 20 mg/kg), animals were subjected to blood collection for analysis of biochemical parameters. Alkaline phosphatase (ALP), aspartate aminotransferase (AST), and alanine aminotransferase (ALT) were all evaluated to assess liver function. Standard procedures and commercially available kits were also used to measure the levels of urea and creatinine (Sattar et al. 2023).

### 2.7 Histopathological Measurements

After being anesthetized, animals were subjected to histopathological analysis, which was conducted after the 28‐day trial period. After being transversely sectioned, the ankle joints of rats were treated with a 10% formalin. Paw tissues were decalcified using methanoic acid, and paraffin blocks were prepared, followed by cutting into thin tissue slides (5 μm). Tissue slides fixed in paraffin blocks were subjected to histological analysis using hematoxylin and eosin (H&E) staining. Tissue changes were identified and examined using light microscopy (Nazar et al. 2024).

### 2.8 Analysis of mRNA Expression Levels of Cytokines

From rat blood, mRNA was extracted using the TRIzol method. To quantify mRNA, the NanoDrop spectrophotometric technique was employed. The cDNA was synthesized using a Thermo Scientific kit, and qPCR was conducted. Two microliters of DNA, 1 μL each of forward and reverse primers, 3 μL of nuclease‐free water, and 6 μL of PCR Master Mix were combined in the PCR procedure. Primer sequences of TNF‐α, NF‐κB, IL‐6, IL‐1β, and IL‐4 were picked from previous studies. Gene expression was analyzed using real‐time qPCR with GAPDH as the housekeeping gene, and relative expression was calculated using the $2^{-∆∆C_{t}}$ method. Primer specificity was confirmed by melt curve analysis, with amplification efficiencies maintained between 90% and 110%. Reagents used for RNA extraction, cDNA synthesis, and qPCR analysis were of analytical grade and obtained from commercial suppliers (Hannan et al. 2023).

### 2.9 Determination of PGE2 Level

PGE2 concentration in serum was measured using ELISA kit E‐EL‐0035 96T, Elabscience Biotechnology Inc. (Wuhan, China) (Mobashar et al. 2020).

### 2.10 Statistical Analysis

GraphPad Prism 8.0 was used. Data were presented as means ± standard error of the mean (SEM). One‐way analysis of variance (ANOVA) followed by Post Tuckey tests was used. A *p*‐value of less than 0.05 was considered significant.

### 2.11 Network Pharmacology

#### 2.11.1 Phytocompounds Dataset Construction

The reported phytocompounds detected in MAO that were previously identified using GC–MS analysis (Aslam et al. 2022). Lipinski's principles were used to filter the phytocompounds reported by GC–MS using SwissADME to determine their “drug‐likeness” physicochemical properties (Gfeller et al. 2014). For phytocompounds, PubChem was used to choose the SMILES (Simplified Molecular Input Line Entry System) (Kim et al. 2023).

#### 2.11.2 Phytocompounds‐Related Target Prediction

The putative targets of MAO phytocompounds reported by GC–MS analysis were predicted using SwissTargetPrediction (STP) with the “ *Homo sapiens* ” preset, which is based on SMILES. SwissTargetPrediction (STP) suggests possible targets for more than 3000 proteins from three species by using a vast library of 370,000 active compounds (Gfeller et al. 2014).

#### 2.11.3 Rheumatoid Arthritis‐Related Target Prediction

The GeneCards database was used to retrieve the targets linked to rheumatoid arthritis. Only proteins from *Homo sapiens* were chosen, and the disease targets from GeneCards were chosen based on score criteria (> 0.9). A variety of data sources, including genomic, proteomic, transcriptomic, genetic, clinical, and functional data, are integrated by GeneCards (Stelzer et al. 2016).

#### 2.11.4 Construction of Venn Diagram

We used VENNY 2.1 to find overlapping targets between MAO phytocompounds targets and rheumatoid arthritis targets. This overlap facilitates the identification of common targets that could be essential to the therapeutic action of drugs and disease pathogenesis (Abid et al. 2026; Oliveros 2007).

#### 2.11.5 Protein–Protein Interaction (PPI) Network Construction

The networks for screened molecular targets were constructed and examined using Cytoscape 3.10.2. The network design, visualization, analysis, target protein identification and their interactions with drugs, and visualization of the diseases and pathways involved are all made possible by this open software platform (Shannon et al. 2003). Protein–protein interactions (PPIs) for hub targets in *Homo sapiens* were predicted using the STRING database. Cytoscape was used to visualize the PPI network once the targets were reintroduced. To rank the most related hubs or nodes in the network, the highest degree of value from the PPI for hub targets was used (Mering et al. 2003; Oraibi et al. 2025).

#### 2.11.6 Gene Ontology and Analysis of Enrichment

The DAVID Database, which comprises Kyoto Encyclopedia of Genes and Genomes (KEGG) pathways and biological processes (BPs), cellular components (CCs), and molecular functions (MFs), was used to conduct gene ontology (GO) enrichment analysis (Dennis et al. 2003; Thomas et al. 2007). Our results were displayed using bubble charts and bar charts using the SRplot platform, proving its value as an online data analysis tool (Jia et al. 2021; Tang et al. 2023). The false discovery rate (FDR < 0.05) applied in GO and KEGG enrichment analyses has now been clearly stated in the Methods section to ensure statistical transparency.

### 2.12 Molecular Docking

Molecular Operating Environment (MOE version 2019.0102) was used to perform molecular docking and scoring calculations on phytocompounds reported by GC–MS analysis for their possible biological targets from MAO and the standard drug piroxicam. The human RAC‐alpha serine/threonine protein kinase AKT1 (PDB ID: 5KCV) at a resolution of 2.70 Å, the human crystal structure of BCL‐2 (PDB ID: 6O0K) at a resolution of 1.62 Å, the human COX‐2 (PDB ID: 5KIR) at a resolution of 2.70 Å, the human MAPK3 (PDB ID: 2ZOQ) at a resolution of 2.39 Å, and the human MMP9 (PDB ID: 5UE4) at a resolution of 1.80 Å were retrieved from Protein Data Bank (Birkinshaw et al. 2019; Kinoshita et al. 2008; Lapierre et al. 2016; Orlando and Malkowski 2016; Scannevin et al. 2017). The MOE Protonate‐3D module handled the protein structure, adding missing properties like protons and charge atoms. The AMBER99 force field was applied to the protein structure for moderate structure. A MOE builder was used to draw the structures of ligands. The AM1‐BBC charges were then determined for each ligand, and the MMFF‐94× force field was used to minimize their structures until an RMS gradient of 0.1 kcal/mol/Å was obtained. Six isolated compounds with 100 conformations each were docked using the technique using MOE's default docking approach, which uses the Triangle Matcher and London dG scoring functions. After docking, conformational sampling was used to choose the best‐docked conformation.

### 2.13 GC/MS Analysis

GC–MS (GC 7890A, MS 5975C; Agilent, USA) analysis of the plant oil was performed to identify the bioactive constituents. The instrument parameters were set as follows: Injection volume was 1 μL, and the capillary column used was DB‐5MS (30 m × 0.25 mm × 0.25 μm). Helium served as the carrier gas at a constant flow rate of 1 mL/min. The oven temperature was initially maintained at 50°C for 2 min, followed by an increase at 7°C/min up to 290°C, where it was held for 1 min. The total run time was 37.286 min, and the injection mode was split (5:1).

The mass spectrometer operated in SCAN mode, with a scan range of m/z (mass to charge ratio) 35–500. The GC–MS analysis was conducted following a previously published method, with minor modifications (Mohan et al. 2026).

## 3 Results

### 3.1 MAO Significantly Reduced Paw Edema in the CFA‐Induced Arthritic Model

**FIGURE 1:**
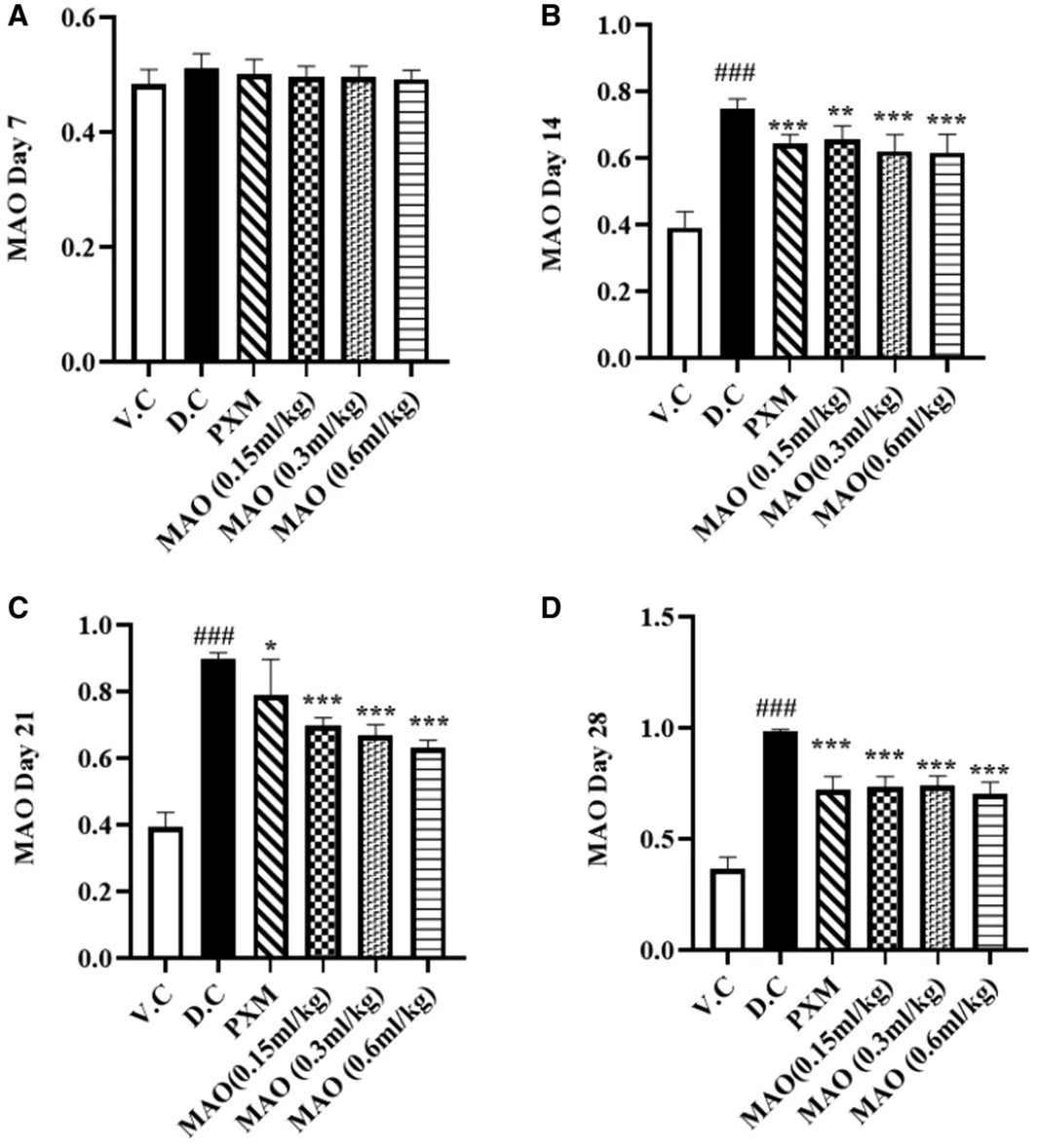
Effect of *M. alternifolia* oil (MAO) on paw edema in CFA‐induced arthritic rats. (A) No significant difference in paw edema was observed among the arthritic and treatment groups on day 7. (B) Piroxicam and MAO significantly attenuated the progression of paw edema on day 14 compared with the arthritic control group. (C) On day 21, treatment with piroxicam and MAO significantly reduced paw edema compared with the arthritic control group. (D) On day 28, piroxicam and MAO treatment markedly decreased paw edema relative to the arthritic control group. Data are presented as mean ± SEM (*n* = 6). Statistical analysis was performed using one‐way ANOVA followed by Tukey's post hoc test. *p < 0.05, **p < 0.01, ***p < 0.001 versus arthritic control group; ^###^ *p* < 0.001 versus normal control group and ns = non‐significant.

No significant change is seen in paw edema between treatment and disease group on day 7 (Figure 1A). Compared with disease group, piroxicam (0.6418 ± 0.01), 0.15 mL/kg MAO (0.655 ± 0.01), 0.3 mL/kg MAO (0.6202 ± 0.02), and 0.6 mL/kg MAO (0.6151 ± 0.02) decrease paw edema on day 14 (Figure 1B). On day 21 (Figure 1C), this inhibitory effect was also present in the groups administered piroxicam (0.789 ± 0.04), 0.15 mL/kg MAO (0.69 ± 0.01), 0.3 mL/kg MAO (0.66 ± 0.01), and 0.6 mL/kg MAO (0.63 ± 0.009). In contrast, paw edema in the disease group was worsened on day 28, and the inhibitory effect was still observed. Piroxicam 10 mg/kg (0.721 ± 0.02), MAO 0.15 mL (0.732 ± 0.01), MAO 0.3 mL/kg (0.7391 ± 0.01), and MAO 0.6 mL/kg (0.702 ± 0.02) significantly reduced paw edema (Figure 1D).

### 3.2 MAO Markedly Decreased Arthritic Score in CFA‐Induced Model

On the 7th day, treatment with piroxicam (3.328 ± 0.077), MAO 0.15 mL/kg (3.417 ± 0.070), MAO 0.3 mL/kg (3.398 ± 0.073), and MAO 0.6 mL/kg (3.377 ± 0.070) showed that no significant difference was detected between treatment and arthritis in terms of arthritis scores (Table 1). However, on the 14th day, there was a significant difference between the arthritic control group (3.463 ± 0.07) and the group given piroxicam (2.393 ± 0.063), MAO 0.15 mL/kg (2.615 ± 0.047), MAO 0.3 mL/kg (2.517 ± 0.039), and MAO 0.6 mL/kg (2.428 ± 0.055). Lastly, on the 28th day, the piroxicam group (2.192 ± 0.046), 0.15 mL/kg MAO (2.390 ± 0.055), 0.3 mL/kg MAO (2.302 ± 0.049), and 0.6 mL/kg MAO (2.230 ± 0.042) all showed a marked reduction in arthritis (Table 1).

**TABLE 1:** Effect of *M. alternifolia* oil (MAO) on arthritic scores in CFA‐induced arthritic rats.

Days	D.C	PXM 10 mg/kg	MAO 0.15 mL/kg	MAO 0.3 mL/kg	MAO 0.6 mL/kg
Mean ± SEM					
7	3.425 ± 0.067	3.328 ± 0.077	3.417 ± 0.070	3.398 ± 0.073	3.377 ± 0.070
14	3.463 ± 0.073^###^	2.393 ± 0.063***	2.615 ± 0.047***	2.517 ± 0.039***	2.428 ± 0.055***
21	3.587 ± 0.046^###^	2.337 ± 0.060***	2.500 ± 0.059***	2.458 ± 0.059***	2.403 ± 0.062***
28	3.935 ± 0.137^###^	2.192 ± 0.046***	2.390 ± 0.055***	2.302 ± 0.049***	2.230 ± 0.042***

*Note:* Data are expressed as mean ± SEM (*n* = 6).

***p < 0.001 versus arthritic control group; ^###^ *p* < 0.001 versus vehicle control group.

### 3.3 MAO Significantly Reduced Histopathological Parameters

**FIGURE 2:**
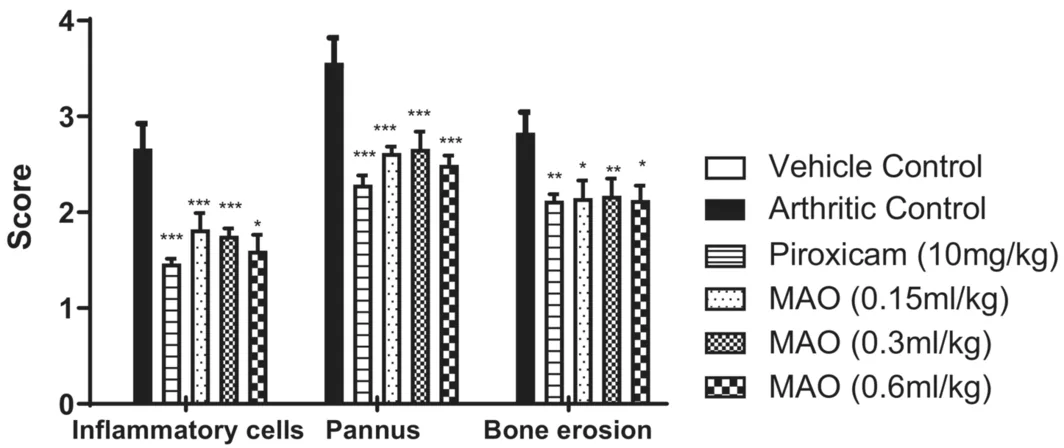
MAO on histopathological parameters in CFA‐induced arthritic rats. Histopathological scoring of inflammatory cell infiltration, pannus formation, and bone erosion in the ankle joints of rats treated with piroxicam and MAO (0.15, 0.3, and 0.6 mL/kg). The vehicle control group exhibited negligible histopathological alterations, resulting in scores close to zero. Data are presented as mean ± SEM (*n* = 6). Statistical analysis was performed using one‐way ANOVA followed by Tukey's post hoc test. *p < 0.05, **p < 0.01, ***p < 0.001 versus arthritic control group.

**FIGURE 3:**
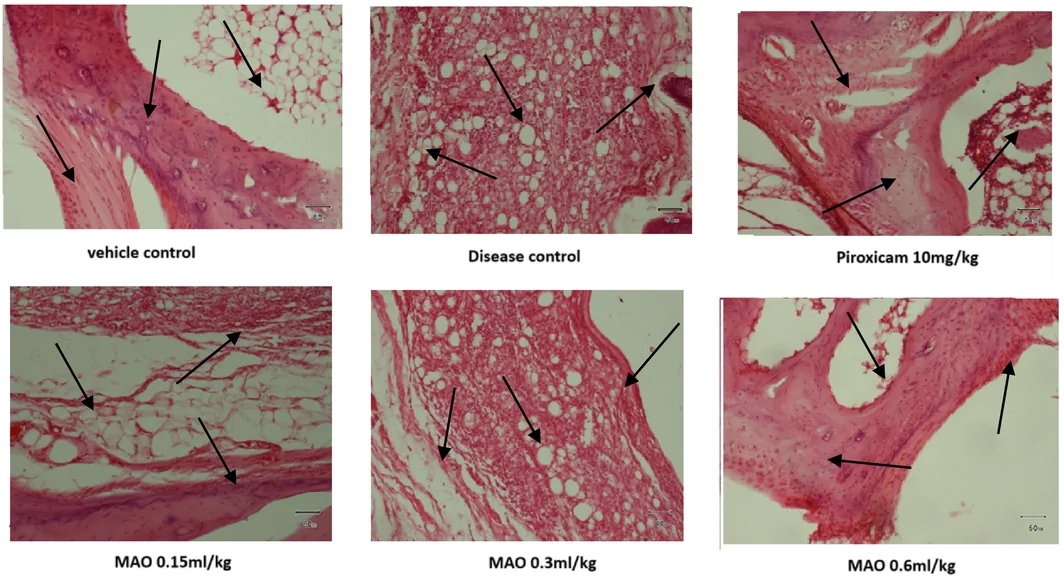
Histopathological investigation of paw of rats. Histopathology of a normal control paw indicates a thin synovial layer enclosed by synovial lining epithelium in absence of inflammatory cell entry as well as pannus formation. In contrast, histopathology of the disease control shows large fibrovascular tissue plaques, granulation tissue, and a large amount of inflammatory cell influx at synovial margin. Paws treated with Piroxicam, MAO 0.15 mL/kg, MAO 0.3 mL/kg, and MAO 0.6 mL/kg showed SM lamina and SM subintima without inflammatory cells or pannus development.

Histopathological examination discloses prominent differences between treatment, normal control, and disease groups. In particular, pannus formation, bone erosion, and infiltration of mononuclear inflammatory cells were observed. While the normal control group showed no evidence of pannus production, bone erosion, and cartilage dysplasia, the disease group showed pannus, cartilage, and bone erosion and mononuclear inflammatory cells. In the piroxicam group, erosion of bone as well as formation of pannus were decreased while MAO 0.15 mL/kg, MAO 0.3 mL/kg, and MAO 0.6 mL/kg significantly reduce bone erosion and pannus formation. A significant decrease in inflammatory cell infiltration was examined with piroxicam (1.463 ± 0.05), MAO 0.15 mL/kg (1.820 ± 0.06), MAO 0.3 mL/kg (1.755 ± 0.03), and MAO 0.6 mL/kg (1.600 ± 0.06) treatment group compared with the arthritic control group (2.66 ± 0.10). A reduction in formation of pannus was noted with piroxicam (2.28 ± 0.09), MAO 0.15 mL/kg (2.617 ± 0.02), MAO 0.3 mL/kg (2.662 ± 0.07), and MAO 0.6 mL/kg (2.493 ± 0.04) compared with the arthritis group (3.56 ± 0.10). Compared with the normal control group, piroxicam (2.12 ± 0.06), MAO 0.15 mL/kg (2.150 ± 0.07), MAO 0.3 mL/kg (2.172 ± 0.07), and MAO 0.6 mL/kg (2.130 ± 0.06) showed a significant reduction in bone erosion (Figure 2). The Figure 3 shows histopathological sections comparing the effects of different treatments on tissue inflammation. The normal control shows normal tissue, while the disease control displays significant damage and inflammation. Piroxicam and varying doses of MAO exhibit a progressive reduction in inflammation, with the highest dose of MAO (0.6 mL/kg) showing near‐normal tissue structure.

### 3.4 MAO Significantly Downregulated mRNA Expression Levels of Pro‐Inflammatory Markers and Upregulated Anti‐Inflammatory Markers

**FIGURE 4:**
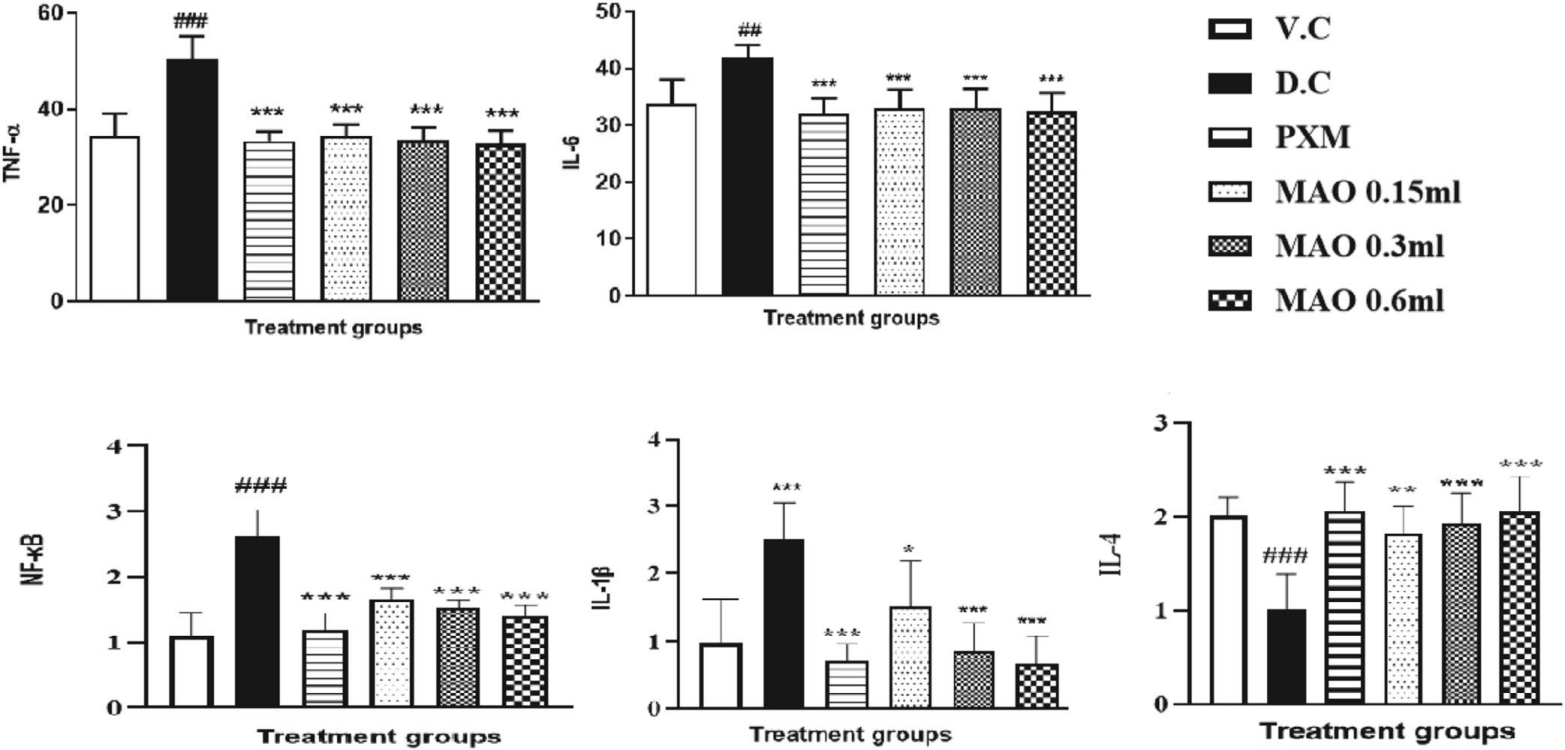
Effect of *M. alternifolia* oil (MAO) on the mRNA expression of pro‐inflammatory and anti‐inflammatory cytokines in CFA‐induced arthritic rats. Treatment with piroxicam and MAO (0.15, 0.3, and 0.6 mL/kg) significantly reduced the expression of TNF‐α, IL‐6, NF‐κB, and IL‐1β, while significantly increasing the expression of IL‐4 compared with the arthritic control group. Data are presented as mean ± SEM (*n* = 6). Statistical analysis was performed using one‐way ANOVA followed by Tukey's post hoc test. *p < 0.05, **p < 0.01, ***p < 0.001 versus arthritic control group; ^##^ *p* < 0.01, ^###^ *p* < 0.001 versus normal control group.

The treatment of rat's paws with Piroxicam (33.29 ± 0.802), MAO 0.15 mL/kg (34.36 ± 0.96), MAO 0.3 mL/kg (33.63 ± 1.02), MAO 0.6 mL/kg (32.90 ± 1.066) notably decrease (*p* < 0.001) TNF‐α level. Simultaneously, reduction in NF‐κB expression was observed with piroxicam (1.183 ± 0.10), MAO 0.15 mL/kg (1.658 ± 0.06), MAO 0.3 mL/kg (1.519 ± 0.05), MAO 0.6 mL/kg (1.398 ± 0.06). With piroxicam 10 mg/kg (31.99 ± 1.131), MAO 0.15 mL/kg (33.06 ± 1.271), MAO 0.3 mL\kg (32.912 ± 1.427), MAO 0.6 mL/kg (32.45 ± 1.270) markedly (*p* < 0.001) decrease in IL‐6 was noticed. Reduction in IL‐1β was seen with piroxicam 10 mg/kg (0.703 ± 0.10), MAO 0.15 mL/kg (1.50 ± 0.27), MAO 0.3 mL/kg (0.85 ± 0.16), MAO 0.6 mL/kg (0.65 ± 0.17). The results also showed that piroxicam (2.062 ± 0.125), MAO 0.15 mL/kg (1.819 ± 0.117), MAO 0.3 mL/kg (1.922 ± 0.131), MAO 0.6 mL/kg (2.051 ± 0.152) upregulated the IL‐4 mRNA expression level and showed significant differences (Figure 4).

### 3.5 MAO Did Not Alter Biochemical Parameters

Piroxicam and MAO had no discernible impact on biochemical markers such as urea level, AST, creatinine, and ALT. More importantly, there was no notable difference in kidney and liver function biomarkers (Table 2).

**TABLE 2:** Effect of *M. alternifolia* oil on liver and kidney function biomarkers in CFA‐induced arthritic rats.

Parameters	V.C	D.C	PXM 10 mg/kg	MAO 0.15 mL/kg	MAO 0.3 mL/kg	MAO 0.6 mL/kg
Mean ± SEM						
AST (U/L)	92.67 ± 0.8433	101.5 ± 0.8466###	94.17 ± 1.078***	92.83 ± 0.7491***	94.00 ± 0.7303***	93.83 ± 1.078**
ALT (U/L)	31.17 ± 0.4014	32.67 ± 0.4944	31.67 ± 0.4216	31.33 ± 0.333	32.17 ± 0.6009	31.17 ± 0.6540
Creatinine (mg/dL)	0.7983 ± 0.0314	0.8118 ± 0.0333	0.8167 ± 0.0288	0.8267 ± 0.0295	0.7917 ± 0.0333	0.7983 ± 0.0200
Urea (mg/dL)	25.83 ± 0.6009	26.38 ± 0.5418	25.54 ± 0.5209	26.05 ± 0.5824	25.70 ± 0.5792	25.67 ± 0.5700

*Note:* Data are expressed as mean ± SEM (*n* = 6).

**p < 0.01 and ***p < 0.001 versus arthritic control group; ^###^ *p* < 0.001 versus vehicle control group.

### 3.6 MAO Restored Hematological Parameters

Hematological parameters were analyzed for piroxicam 10 mg/kg, MAO 0.15 mL/kg, MAO 0.3 mL/kg, MAO 0.6 mL/kg compared to the disease control group. A significant increase in red blood cell count was observed in the above treatment group as compared to the arthritic control group. MAO treatment groups showed restoration of Hb count compared to the Hb level in the arthritic control group. The findings also showed that white blood counts increased and platelet counts returned to normal in the treatment groups as compared with the arthritic control. The arthritic control group showed suppression of the RBC count (5.533 ± 0.340) *p* < 0.001 and the Hb count (11.75 ± 0.210) *p* < 0.001 when compared to the normal control and treatment groups. In the arthritic group's WBCs (15.04 ± 0.267) and platelets (1452 ± 16.15) were higher than those of the normal control group (Table 3).

**TABLE 3:** Effect of *M. alternifolia* oil (MAO) on hematological parameters in CFA‐induced arthritic rats.

Parameters	V.C	D.C	PXM 10 mg/kg	MAO 0.15 mL/kg	MAO 0.3 mL/kg	MAO 0.6 mL/kg
Mean ± SEM						
Hb (g/dL)	14.50 ± 0.1415	11.75 ± 0.2106^###^	13.58 ± 0.2734**	13.57 ± 0.3226**	13.56 ± 0.3300**	13.67 ± 0.3589***
RBC (10^6^/Ul)	8.275 ± 0.2903	5.533 ± 0.3401^###^	7.595 ± 0.2645***	7.515 ± 0.2811***	7.597 ± 0.2591***	7.668 ± 0.2501***
WBC (10^3^/Ul)	9.929 ± 0.3768	15.04 ± 0.2673^###^	12.48 ± 0.2894***	12.54 ± 0.1392***	12.62 ± 0.1821***	12.79 ± 0.00760***
Platelets (10^3^/Ul)	802.7 ± 25.71	1452 ± 16.15^###^	1045 ± 35.12***	945.7 ± 16.11***	929.7 ± 17.26***	914.5 ± 17.30***

*Note:* Data are presented as mean ± SEM (*n* = 6).

**p < 0.01 and ***p < 0.001 versus arthritic control group; ^###^ *p* < 0.001 versus vehicle control group.

### 3.7 MAO Significantly Reduced PGE2

**FIGURE 5:**
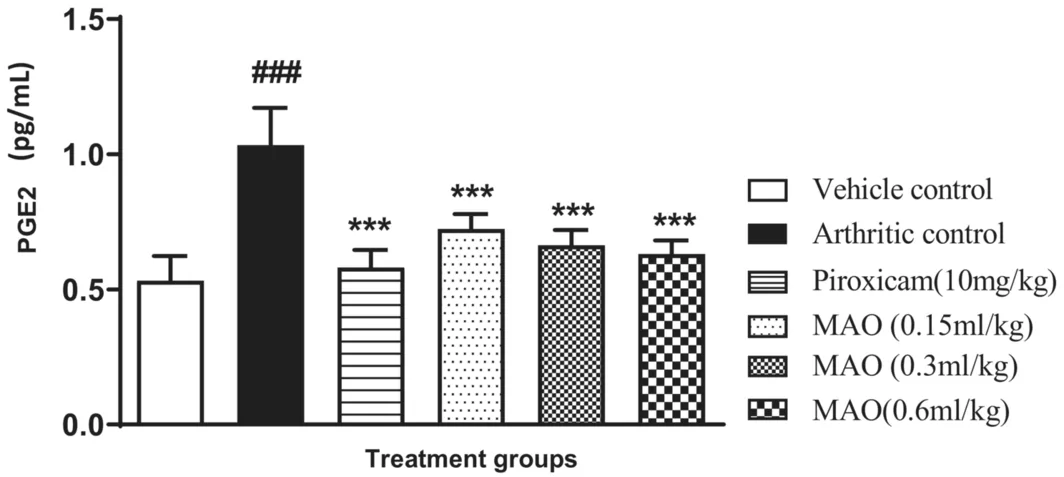
Effect of MAO on serum PGE2 levels in CFA‐induced arthritic rats. Treatment with piroxicam and MAO (0.15, 0.3, and 0.6 mL/kg) significantly reduced PGE2 levels compared with the arthritic control group. Data are presented as mean ± SEM (*n* = 6). Statistical analysis was performed using one‐way ANOVA followed by Tukey's post hoc test. ***p < 0.001 versus arthritic control group; ^###^ *p* < 0.001 versus normal control group.

The results indicated that the arthritic control group's PGE2 value (pg/mL) was substantially higher (*p* < 0.001) than that of the negative group. In addition, the PGE2 levels of the MAO 0.15 mL/kg (0.722 ± 0.022), MAO 0.3 mL/kg (0.662 ± 0.02), MAO 0.6 mL/kg (0.630 ± 0.02), and Piroxicam 10 mg/kg (0.581 ± 0.026) significantly decreased with *p* < 0.001 when compared to the arthritic control (Figure 5).

### 3.8 Physicochemical Properties of Phytocompounds

A total of 34 phytocompounds were reported by GC–MS analysis in the MAO (Malik and Upadhyay 2022). Lipinski's rules (molecular weight ≤ 500 g/mol; Moriguchi octanol–water partition coefficient ≤ 4.15; number of nitrogen or oxygen ≤ 10; number of NH or OH ≤ 5) were fulfilled by all 34 compounds, and all phytocompounds met the SwissADME standard of “Abbott Bioavailability Score (> 0.1)”. All phytocompounds' TPSA (Topological Polar Surface Area) values were also approved (Tables 4 and 5).

**TABLE 4:** The phytocompounds identified in *M. alternifolia* oil, their chemical class and canonical smiles.

No.	Compounds	Chemical class	Canonical smiles
1	Camphene	Terpenoid	CC1(C2CCC(C2)C1=C)C
2	Sabinene	Terpenoid	CC(C)[C@]12CCC(=C)[C@H]1C2
3	Limolene	Terpenoid	CC1=CC[C@H](C(C)=C)CC1
4	α‐phellendrene	Terpenoid	CC1=CCC(C=C1)C(C)C
5	β–pinene	Terpenoid	CC1(C2CCC(=C)C1C2)C
6	Verbenone	Terpenoid	CC1=CC(=O)[C@@H]2C[C@H]1C2(C)C
7	Trans‐β‐ocimene	Terpenoid	CC(=CC/C=C(\C)/C=C)C
8	α‐pinene	Terpenoid	CC1=CCC2CC1C2(C)C
9	Terpinolene	Terpenoid	CC1=CCC(=C(C)C)CC1
10	Linalool	Terpenoid	CC(O)(C=C)CCC=C(C)C
11	α‐pinene‐epoxide	Terpenoid	CC1([C@H]2C[C@@H]1C3(C(C2)O3)C)C
12	Neo‐allo‐ocimene	Terpenoid	[H]/C(C([H])([H])[H])=C(C([H])([H])[H])/C([H])=C([H])/C([H])=C(C([H])([H])[H])/C([H])([H])[H]
13	Trans‐myroxide	Terpenoid	C/C(=C\CC1C(O1)(C)C)/C=C
14	Cis‐tagetone	Monoterpene ketone	CC(C)CC(=O)/C=C(/C)\C=C
15	Camphor	Alcohol derivative	CC1(C2CCC1(C(=O)C2)C)C
16	Terpineol	Terpenoid	CC1=CCC(CC1)C(C)(C)O
17	Viridiflorol	Sesquiterpene alcohol	C[C@@H]1CC[C@H]2[C@@H]1[C@H]3[C@H](C3(C)C)CC[C@]2(C)O
18	Terpinene‐4‐ol	Terpenoid	CC1=CCC(CC1)(C(C)C)O
19	β‐caryophyllene	Phenylpropanoid	C/C/1=C\CCC(=C)[C@H]2CC([C@@H]2CC1)(C)C
20	γ‐terpinene	Terpenoid	CC1=CCC(=CC1)C(C)C
21	Trans‐pinocarveol	Phenylpropanoid	CC1([C@H]2C[C@@H]1C(=C)[C@@H](C2)O)C
22	α‐terpinene	Terpenoid	CC1=CC=C(CC1)C(C)C
23	Trans‐ocimenone	Monoterpene	CC(=CC(=O)/C=C(\C)/C=C)C
24	Aromadendrene	Terpenoid	CC1CCC2C1C3C(C3(C)C)CCC2=C
25	Citronellyl formate	Alcohol derivative	CC(CCC=C(C)C)CCOC=O
26	Isopiperitenone	Phenylpropanoid	CC1=CC(=O)C(CC1)C(=C)C
27	(E)‐caryophyllene	Phenylpropanoid	C/C(CC[C@@H]1[C@@H]2CC1(C)C)=C\CCC2=C
28	Cyclohexanol	Alcohol derivative	C1CCC(CC1)O
29	Citronellyl propionate	Alcohol derivative	CCC(=O)OCCC(C)CCC=C(C)C
30	Globulol	Terpenoid	C[C@@H]1CC[C@@H]2[C@@H]1[C@H]3[C@H](C3(C)C)CC[C@@]2(C)O
31	1,8 Cineole	Terpenoid	CC1(C2CCC(O1)(CC2)C)C
32	β‐Terpineol	Terpenoid	CC1(O)CCC(C(C)=C)CC1
33	Ρ‐Cymene	Terpenoid	CC1=CC=C(C=C1)C(C)C
34	Terpineol	Terpenoid	C\C1=C\CC(CC1)C(O)(C)C

**TABLE 5:** Physicochemical properties of phytocompounds for cell membrane permeability and oral bioavailability.

No.	Compounds	Lipinski rules				Lipinski's violations	Bioavailability score	TPSA (A^2^)
		MW	HBA	HBD	MLogP			
		< 500	< 10	≤ 5	≤ 4.15	≤ 1	> 0.1	< 140
1	Camphene	136.23	0	0	4.29	1	0.55	0
2	Sabinene	136.23	0	0	4.29	1	0.55	0
3	Limolene	136.23	0	0	3.27	0	0.55	0
4	α‐phellendrene	136.23	0	0	3.27	0	0.55	0
5	β‐pinene	136.23	0	0	4.29	1	0.55	0
6	Verbenone	150.22	1	0	2.2	0	0.55	17.07
7	Trans‐β‐ocimene	136.23	0	0	3.56	0	0.55	0
8	α‐pinene	136.23	0	0	4.29	1	0.55	0
9	Terpinolene	136.23	0	0	3.27	0	0.55	0
10	Linalool	154.25	1	1	2.59	0	0.55	20.23
11	α‐pinene‐epoxide	152.23	1	0	2.45	0	0.55	12.53
12	Neo‐allo‐ocimene	136.23	0	0	2.45	0	0.55	0
13	Trans‐myroxide	152.23	1	0	2.2	0	0.55	12.53
14	Cis‐tagetone	152.23	1	0	2.49	0	0.55	17.07
15	Camphor	152.23	1	0	2.3	0	0.55	17.07
16	Terpineol	154.25	1	1	2.3	0	0.55	20.23
17	Viridiflorol	222.37	1	1	3.81	0	0.55	20.23
18	Terpinene‐4‐ol	154.25	1	1	2.3	0	0.55	20.23
19	β‐caryophyllene	204.35	0	0	4.63	1	0.55	0
20	*γ*‐terpinene	136.23	0	0	3.27	0	0.55	0
21	Trans‐pinocarveol	152.23	1	1	2.3	0	0.55	20.23
22	α‐terpinene	136.23	0	0	3.27	0	0.55	0
23	Trans‐ocimenone	150.22	1	0	2.4	0	0.55	17.07
24	Aromadendrene	204.35	0	0	5.65	1	0.55	0
25	Citronellyl formate	184.28	2	0	2.76	0	0.55	26.3
26	Isopiperitenone	150.22	1	0	2.1	0	0.55	17.07
27	(E)‐caryophyllene	204.35	0	0	4.63	1	0.55	0
28	Cyclohexanol	100.16	1	1	1.14	0	0.55	20.23
29	Citronellyl propionate	212.33	2	0	3.32	0	0.55	26.3
30	Globulol	222.37	1	1	3.81	0	0.55	20.23
31	1,8 Cineole	154.25	1	0	2.45	0	0.55	9.23
32	β‐Terpineol	154.25	1	1	2.3	0	0.55	20.23
33	*p*‐Cymene	134.22	0	0	4.47	1	0.55	0
34	Terpineol	154.25	1	1	2.3	0	0.55	20.23

### 3.9 Targets Related With Phytocompounds

34 phytocompounds were linked to 247 main targets that were predicted from STP. Each of these active compounds was linked to several targets. This suggests that when MAO is used as an anti‐rheumatic drug, it may have a synergistic effect on a variety of targets.

### 3.10 Overlapping Targets Between Rheumatoid Arthritis‐Linked Targets and Phytocompound‐Linked Targets

**FIGURE 6:**
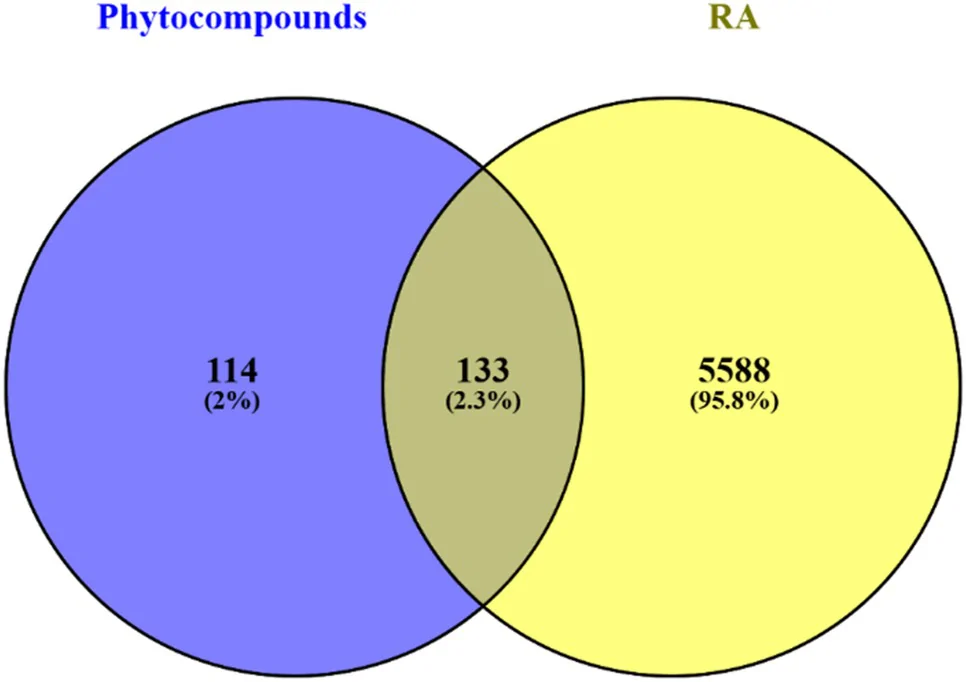
Overlapped targets of phytocompounds (247 targets) and rheumatoid arthritis‐associated targets (5721 targets).

From public databases, 5721 targets for rheumatoid arthritis were found. Among the 5721 targets linked to rheumatoid arthritis and the 247 targets linked to phytocompounds, the Venn diagram showed 133 overlapping targets (Figure 6).

### 3.11 Protein–Protein Interaction (PPI) Network Construction

**FIGURE 7:**
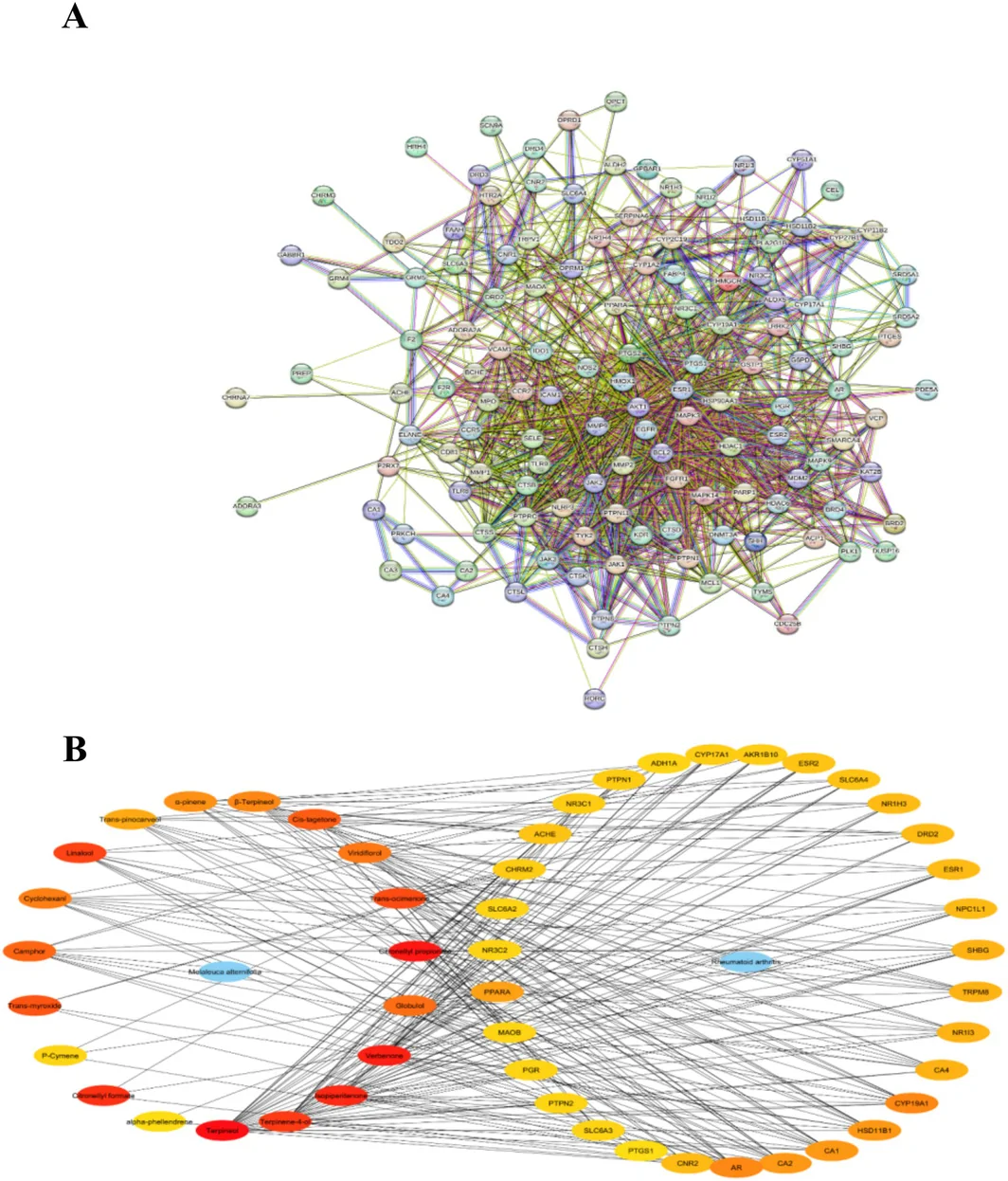
Network pharmacology‐based analysis of compounds and multiple targets for anti‐rheumatic activity. (A) PPI networks (132 nodes and 1144 edges) and (B) Network diagram of phytocompounds and with top 50 targets. The reddish‐yellow ellipse indicates MAO phytocompounds, yellow‐orange indicates the rheumatoid arthritis targets, and nodes indicate the pathways.

132 unique targets out of 133 overlapping targets from the STRING analysis were directly linked to the development and occurrence of rheumatoid arthritis, showing 1144 edges and 132 nodes (Figure 7A). The STRING network was then subjected to PPI network analysis using Cytoscape 3.10.2, which showed a network of interactions. The AKT1 target was regarded as a hub target and had the highest degree in PPI networks (Score: 156) (Table 6). The targeted genes may be important targets because the highest degree suggests a substantial link between them (Figure 7B). These genes, specifically AKT1, BCL‐2, PTGS2 (COX‐2), MAPK3, and MMP9, were selected for molecular docking studies after these results were compared with those provided by enrichment analysis. These genes were found to be the primary anti‐rheumatic targets of MAO.

**TABLE 6:** Top 50 PPI networks and their degree value.

No	Target	Degree	No.	Target	Degree
1	AKT1	156	26	PTPN11	50
2	BCL2	118	27	CYP1A2	48
3	PTGS2	116	28	IDO1	48
4	MAPK3	116	29	HDAC1	48
5	MMP9	112	30	CCR5	48
6	ESR1	102	31	NLRP3	46
7	EGFR	98	32	MAOA	46
8	HSP90AA1	96	33	CTSB	46
9	JAK2	78	34	MCL1	46
10	NR3C1	72	35	CYP19A1	46
11	ICAM1	72	36	CCR2	46
12	MMP2	70	37	ESR2	44
13	MAPK14	68	38	PTGS1	44
14	PARP1	64	39	HMGCR	44
15	PTPRC	64	40	MMP1	42
16	KDR	60	41	NOS2	42
17	VCAM1	60	42	SELE	42
18	PGR	58	43	CTSS	42
19	AR	58	44	CYP17A1	42
20	PPARA	58	45	HDAC6	40
21	MDM2	58	46	OPRM1	40
22	TLR9	56	47	CYP2C19	40
23	HMOX1	56	48	FGFR1	38
24	JAK1	52	49	DRD2	38
25	MPO	52	50	SMARCA4	36

### 3.12 Genome Enrichment Analysis

**FIGURE 8:**
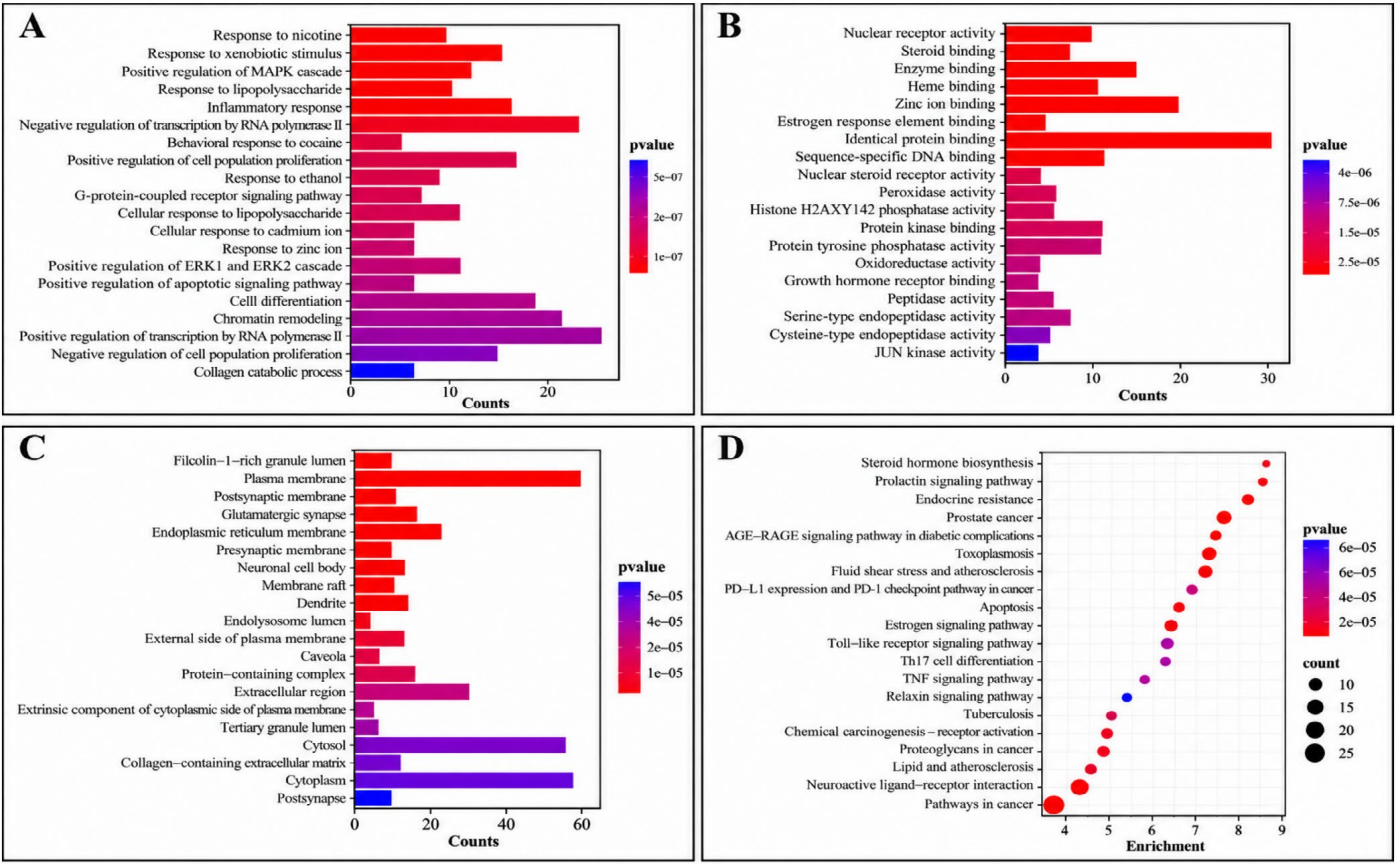
SRplot plot: Bubble and bar graphs that show the functional annotation and possible routes of phytocompounds for the treatment of rheumatoid arthritis. (A) Biological process, (B) molecular functions, and (C) cellular components. The size of each bubble represents the number of genes enriched in each pathway. More genes involved in the pathway are indicated by a larger bubble. The enrichment value was taken on the *x*‐axis. (D) KEGG pathway: The top 20 items of KEGG pathway. The adjusted *p* value for every GO phrase was indicated by the color of each bar. In both the bubble and bar plots, the bluer the term's adjusted *p* value, the greater its enrichment.

The PPI network connects 132 unique rheumatoid arthritis‐related targets based on their routes and severity. The biological roles of MAO targets were identified by functional annotation and enrichment analysis (Table 6). The biological process (BP), cellular composition (CC), molecular function (MF), and KEGG pathway entries were determined for overlapped targets by GO enrichment analysis. KEGG pathway analysis was carried out to determine the important signaling pathways connected to MAO's anti‐rheumatic impact (Kanehisa 2019; Kanehisa and Goto 2000). Rheumatoid arthritis was directly linked to 20 KEGG pathway signaling pathways, indicating that these 20 signaling pathways may be the MAO's most notable defenses against RA (Kanehisa et al. 2023) (Figure 8A–D).

### 3.13 Molecular Docking of 5 Targets

The AKT1 target was linked with one phytocompound (citronellyl propionate), the BCL‐2 target was linked with one phytocompound (citronellyl formate), the PTGS2 (COX‐2) target was related to three phytocompounds (linalool, trans‐myroxide, and citronellyl propionate), the MAPK3 target was related to two phytocompounds (verbenone and isopiperitenone), and the MMP9 target was related to one phytocompound (citronellyl propionate). The citronellyl propionate inhibits the three molecular targets in rheumatoid arthritis disease. The affinity of the target protein(s) and phytocompound(s) was verified using molecular docking.

### 3.14 Computational Analysis

**FIGURE 9:**
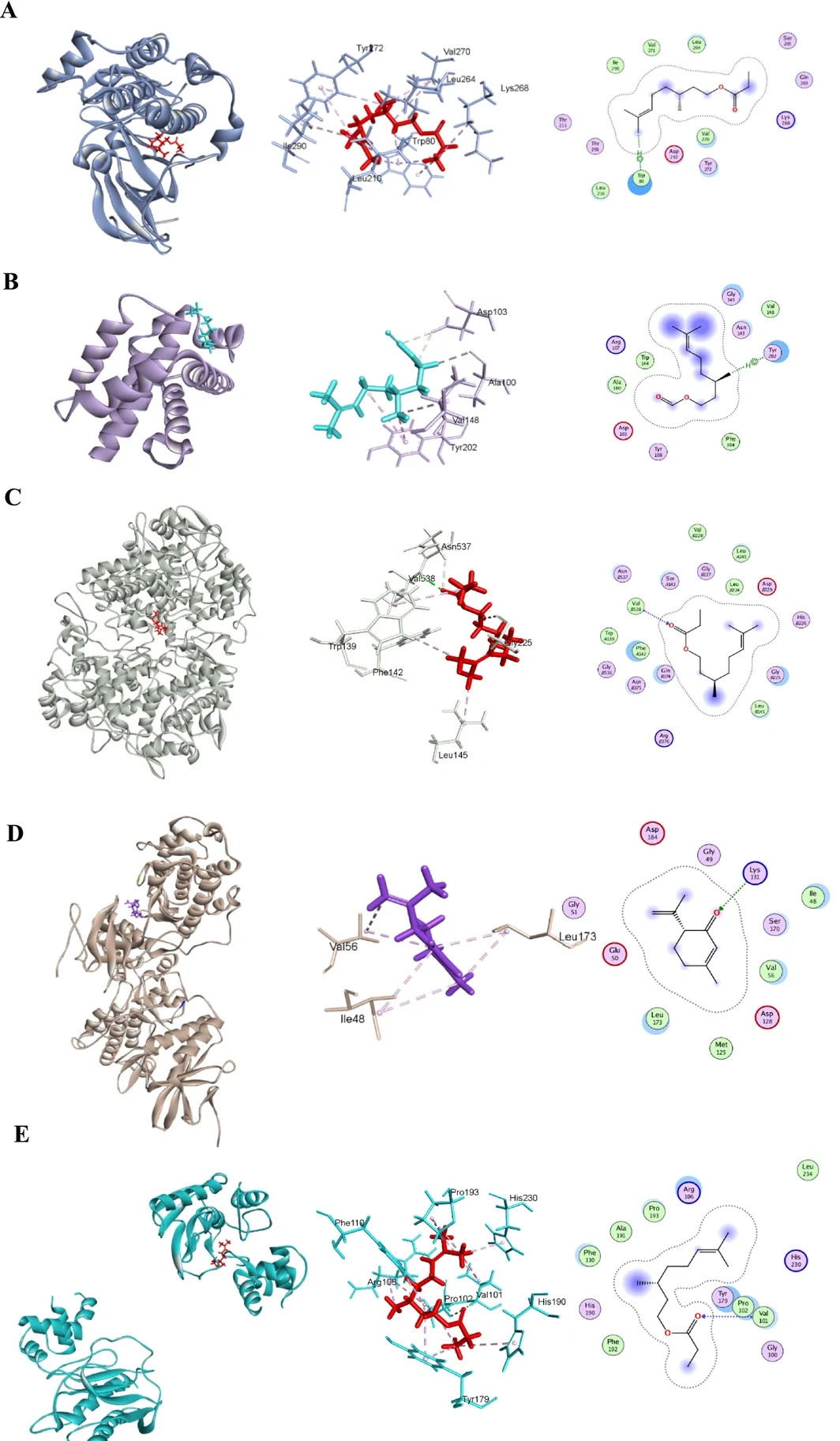
The docked complexes of five targets along with their highest affinity phytocompounds. (A) AKT1 docked with citronellyl propionate; (B) BCL‐2 docked with citronellyl formate; (C) COX‐2 docked with citronellyl propionate; (D) MAPK3 docked with isopiperitenone; and (E) MMP9 docked with citronellyl propionate.

The docking data provide insights into the binding interactions of various phytocompounds with multiple protein targets, including AKT1 (PDB ID: 5KCV), BCL‐2 (PDB ID: 6O0K), COX‐2 (PDB ID: 5IKR), MAPK3 (PDB ID: 2ZOQ), and MMP9 (PDB ID: 5UE4). The binding affinity (S score) and root‐mean‐square deviation (RMSD) values were analyzed to determine the stability and strength of these interactions. For AKT1, Citronellyl propionate exhibited a binding affinity of −5.87 kcal/mol with an RMSD of 0.82 Å. It interacted with TRP 80 (A) through an H‐pi bond at a distance of 4.71 Å. In comparison, the standard drug Piroxicam demonstrated a stronger binding affinity of −6.78 kcal/mol with an RMSD of 2.02 Å. It formed a pi‐H interaction with GLN 79 (A) at a distance of 3.72 Å (Table 7). Similarly, the docking results for BCL‐2 (PDB ID: 6O0K) revealed that Citronellyl formate had a binding affinity of −4.50 kcal/mol and an RMSD of 1.17 Å. It interacted with TYR 202 (A) via an H‐pi bond at a distance of 3.78 Å. In contrast, Piroxicam exhibited a binding affinity of −5.11 kcal/mol with an RMSD of 1.75 Å, forming an H‐donor interaction with ASP 103 (A) at a distance of 3.37 Å (Table 8). For COX‐2 (PDB ID: 5IKR), Citronellyl propionate displayed a binding affinity of −6.21 kcal/mol with an RMSD of 1.67 Å. It formed an H‐acceptor bond with VAL 538 (B) at a distance of 3.01 Å. Trans‐myroxide and Linalool exhibited binding affinities of −5.21 kcal/mol and −5.61 kcal/mol, respectively, interacting with ASN 537 (B) and ASN 375 (B) through H‐acceptor and H‐donor bonds. Piroxicam, as the standard drug, showed the highest binding affinity of −6.49 kcal/mol with an RMSD of 1.80 Å. It interacted with ASN 375 (B) and ARG 376 (A) through H‐acceptor and pi‐cation interactions at distances of 2.83 Å and 4.21 Å, respectively (Table 9). For MAPK3 (PDB ID: 2ZOQ), Verbenone exhibited a binding affinity of −4.79 kcal/mol with an RMSD of 1.69 Å. It interacted with CYS 183 (A) and SER 170 (A) through H‐donor and H‐acceptor interactions at distances of 3.63 Å and 2.98 Å, respectively. Isopiperitenone had a binding affinity of −4.85 kcal/mol and formed an H‐acceptor interaction with LYS 131 (A) at a distance of 3.27 Å. Piroxicam displayed the highest binding affinity of −6.74 kcal/mol with an RMSD of 2.22 Å. It formed H‐donor, H‐acceptor, and pi‐cation interactions with ASP 184 (A), LYS 131 (A), and LYS 168 (A) at distances of 3.12 Å, 2.96 Å, and 3.99 Å, respectively (Table 10). In the docking analysis of MMP9 (PDB ID: 5UE4), Citronellyl propionate demonstrated a binding affinity of −6.21 kcal/mol with an RMSD of 2.09 Å. It formed an H‐acceptor bond with VAL 101 (B) at a distance of 3.39 Å. Piroxicam, the standard drug, exhibited a binding affinity of −6.03 kcal/mol with an RMSD of 0.88 Å. It interacted with ALA 191 (B) and TYR 50 (B) through H‐donor and H‐pi bonds at distances of 3.21 Å and 3.42 Å, respectively (Table 11). These results exhibited that active constituents of MAO bind stably with five target proteins and function as an anti‐rheumatic agent. Molecular docking analysis demonstrated that citronellyl propionate (Figure 9A), citronellyl formate (Figure 9B), citronellyl propionate (Figure 9C) isopiperitenone (Figure 9D) and citronellyl propionate (Figure 9E) showed stronger binding energies with the target protein that are comparable to the standard drug. All the drug candidates showed hydrogen bond, Pi–pi‐stacked, and van der Waals interactions with the receptor proteins.

**TABLE 7:** In silico analysis of potential phytocompounds targeting AKT1 binding sites.

Compound	S score (kcal/mol)	RMSD (Å)	Atom of compounds	Atom of receptors	Residue of receptor	Type of interaction bond	Distance (Å)
AKT1 (PDB ID: 5KCV)							
Citronellyl propionate	−5.87	0.82	C‐32	5‐ring	TRP 80 (A)	H‐pi	4.71
Piroxicam	−6.78	2.02	6‐ring	CB	GLN 79 (A)	pi‐H	3.72

**TABLE 8:** In silico analysis of potential phytocompounds targeting BCL‐2 binding sites.

Compound	S score (kcal/mol)	RMSD (Å)	Atom of compounds	Atom of receptors	Residue of receptor	Type of interaction bond	Distance (Å)
BCL‐2 (PDB ID: 6O0K)							
Citronellyl formate	−4.50	1.17	C‐1	6‐ring	TYR 202 (A)	H‐pi	3.78
Piroxicam	−5.11	1.75	O‐1	OD2	ASP 103 (A)	H‐donor	3.37

**TABLE 9:** In silico analysis of potential phytocompounds targeting COX‐2 binding sites.

Compound	S score (kcal/mol)	RMSD (Å)	Atom of compounds	Atom of receptors	Residue of receptor	Type of interaction bond	Distance (Å)
COX‐2 (PDB ID: 5IKR)							
Citronellyl propionate	−6.21	1.67	O‐9	N	VAL 538 (B)	H‐acceptor	3.01
Trans‐myroxide	−5.21	0.94	O‐14	CA	ASN 537 (B)	H‐acceptor	3.31
Linalool	−5.61	1.30	O‐6	O	ASN 375 (B)	H‐donor	3.31
Piroxicam	−6.49	1.80	O‐24 6‐ring	N NH2	ASN 375 (B) ARG 376 (A)	H‐acceptor pi‐cation	2.83 4.21

**TABLE 10:** In silico analysis of potential phytocompounds targeting MAPK3 binding sites.

Compound	S score (kcal/mol)	RMSD (Å)	Atom of compounds	Atom of receptors	Residue of receptor	Type of interaction bond	Distance (Å)
MAPK3 (PDB ID: 2ZOQ)							
Verbenone	−4.79	1.69	C‐6 O‐9	SG OG	CYS 183 (A) SER 170 (A)	H‐donor H‐acceptor	3.63 2.98
Isopiperitenone	−4.85	1.44	O‐9	NZ	LYS 131 (A)	H‐acceptor	3.27
Piroxicam	−6.74	2.22	O‐1 O‐16 6‐ring	OD2 NZ NZ	ASP 184 (A) LYS 131 (A) LYS 168 (A)	H‐donor H‐acceptor pi‐cation	3.12 2.96 3.99

**TABLE 11:** In silico analysis of potential phytocompounds targeting MMP9 binding sites.

Compound	S score (kcal/mol)	RMSD (Å)	Atom of compounds	Atom of receptors	Residue of receptor	Type of interaction bond	Distance (Å)
MMP9 (PDB ID: 5UE4)							
Citronellyl propionate	−6.21	2.09	O‐9	CA	VAL 101 (B)	H‐acceptor	3.39
Piroxicam	−6.03	0.88	C‐11 C‐32	O 6‐ring	ALA 191 (B) TYR 50 (B)	H‐donor H‐pi	3.21 3.42

### 3.15 GCMS Analysis

GC/MS analysis of plant's oil reported the presence of anti‐inflammatory and antirheumatic compounds. The identified constituents with their retention time, total percentage, and quality are shown in Table 12.

**TABLE 12:** List of identified components of *M. alternifolia* oil from mass chromatograms.

Name of identified compound	Quality	Area %	Retention time (min)
Bicyclo[3.1.1]heptan‐3‐ol, 6,6‐dimethyl‐2‐methylene‐, [1S‐ (1.alpha., 3.alpha., 5.alpha.)]	81	1.70	6.905
Bicyclo[3.3.1]nonan‐3‐ol, 7‐methylene‐	55	1.70	6.905
Isopinocarveol	45	1.70	6.905
Pinocarvone	76	1.03	6.960
Terpinen‐4‐ol	93	28.02	8.019
(‐)‐Myrtenol	70	8.093	2.61
Bicyclo[3.1.1]hept‐2‐ene‐2‐methanol, 6,6‐dimethyl‐	58	8.093	2.61
2H‐Inden‐2‐one, 1,3,3a,4,7,7a‐hexahydro‐, trans‐	58	8.093	2.61
Bicyclo[3.1.1]hept‐2‐ene‐2‐carboxaldehyde, 6,6‐dimethyl	98	8.125	1.97
Hexanal, 4,4‐dimethyl‐	43	8.180	0.37
2H‐Pyran‐3‐ol, tetrahydro‐3‐methyl‐6‐propyl‐, acetate	38	8.180	0.37
3‐Acetyloxypiperidin‐2‐one	38	8.180	0.37
2‐Cyclohexen‐1‐ol, 2‐methyl‐5‐(1‐methylethenyl)‐, cis‐	60	8.208	0.29
Carveol	52	8.208	0.29
Carvone	95	8.313	0.85
D‐Carvone	93	8.313	0.85
(3‐Fluorophenyl) methanol, n‐propyl ether	38	8.495	8.495
3‐(3,5‐Dimethyl‐1H‐pyrazol‐1‐yl)propylamine	27	8.495	8.495
6,6‐Dimethylhepta‐2,4‐diene	27	8.495	8.495
trans‐2,3‐Epoxydecane	89	8.556	1.06
1‐Methylcyclooctanol	22	8.556	1.06
Vinyl 2‐ethyl‐2,5‐dimethylhexanoate	22	8.556	1.06
Benzene, 1‐methoxy‐4‐(1‐propenyl)‐, (Z)‐	94	8.673	5.62
Anethole	94	8.673	5.62
Anethole, trans	96	8.707	0.98
6‐Dodecanone	35	8.783	2.98
7‐Pentadecanone	35	8.783	2.98
7‐Tridecanone	27	8.783	2.98
trans‐Ascaridol glycol	72	8.854	3.83
1H‐Pyrazole‐4‐carboxylic acid, 3‐amino‐	50	8.854	3.83
2,4‐Hexadiene, 2,5‐dimethyl‐	50	8.897	0.65
2,4‐Hexadiene, 3,4‐dimethyl‐, (E, Z)‐	45	8.897	0.65
2,4‐Hexadienoic acid, ethyl ester, (2E,4E)‐	43	8.897	0.65
2‐Thiophenecarboxamide, N‐hydroxy‐	47	8.928	0.53
2H‐Pyran‐2‐carboxaldehyde, 3,4‐dihydro‐2,5‐dimethyl‐	46	8.928	0.53
1‐Cyclohexanone, 3‐butyl‐3‐methyl	43	8.928	0.53
2‐Pyrazoline, 1,3,4‐trimethyl	46	9.051	0.61
Methyl‐(3Z)‐hexen‐2‐one,5	35	9.051	0.61
Thiophene, 2,3‐dimethyl	30	9.051	0.61
Tricyclo [4.4.0.0 (2,8)] decan‐3‐ol	25	9.084	1.03
Spiro[5.6]dodecane	15	9.084	1.03
Octanoic acid, 7‐hydroxy‐3,7‐dimethyl‐, methyl ester	14	9.084	1.03
1‐Aminocyclopropanecarboxylic acid, N‐dimethylaminomethylene‐, butyl ester	43	9.189	0.43
1,3‐Cyclohexanediol, 5‐methyl‐2‐nitro‐, monoacetate (ester), [1 s‐(1.alpha., 2.beta., 3.alpha., 5.alpha.)]‐	43	9.189	0.43
trans‐Arbusculone	43	9.189	0.43
2‐n‐Hexylcyclopentanone	47	9.258	0.79
1‐Pentylpyrrolidine	43	9.258	0.79
2‐Butenal, 2‐methyl‐, (E)	43	9.258	0.79
6‐Dodecanol acetate	38	9.324	0.19
1,3‐Cyclohexanediamine	37	9.342	0.19
Hexacosyl propyl ether	32	9.342	0.19
Piperitol, cis‐	47	9.372	0.48
2‐Butenal, 3‐methyl‐	46	9.372	0.48
2‐Cyclohexen‐1‐ol, 3‐methyl‐6‐(1‐methylethyl)‐, cis‐	43	9.372	0.48
Linalool acetate, dihydro‐	47	9.453	2.42
3,5‐Heptadien‐2‐one, 6‐methyl‐, (E)‐	43	9.453	2.42
Phenol, 3‐amino‐	43	9.453	2.42
Oxan‐3‐ol, acetate	38	9.673	5.44
2‐Butanone, 1‐(2‐furanyl)‐3‐methyl‐	30	9.673	5.44
D‐Fenchone	14	9.673	5.44
4‐Methyl‐4‐vinylbutyrolactone	53	9.716	0.99
4‐Hepten‐3‐one, 2,5,6‐trimethyl	53	9.716	0.99
2(1H)‐Pyridinone, 5‐hydroxy‐	38	9.716	0.99
S‐(2‐((1R,4S)‐4‐methyl‐2‐oxocyclohexyl)propan‐2‐yl) ethanethioate, rel	53	9.774	1.22
Phosphonic acid, (2‐hydroxybicyclo[2.2.1]hept‐5‐en‐2‐yl)‐, dimethyl ester, endo‐	38	9.774	1.22
Cyclopentanecarboxylic acid, allyl ester	35	9.774	1.22
Cyclohexanol, 3,3,5‐trimethyl‐, acetate, cis	35	9.881	4.45
3,3,5‐Trimethylcyclohexyl methylphosphonofluoridoate	35	9.881	4.45
Phenol, 4‐amino‐	30	9.881	4.45
2‐Acetyl‐1,4,5,6‐tetrahydropyridine	46	9.918	1.44
2‐Amino‐5‐methyl‐4‐oxo‐3,4‐dihydropyrimidine	38	9.918	1.44
Imidazole, 2‐aminocarbonyl‐1‐methyl	35	9.918	1.44
11‐Oxa‐tricyclo[4.4.1.0(1,6)]undecan‐2‐ol	22	9.981	0.27
Butanoic acid, 2‐methyl‐5‐oxo‐1‐cyclopenten‐1‐yl ester	18	9.981	0.27
2‐Thiophenecarboxylic acid, isopropyl ester	14	9.981	0.27
Fumaric acid, ethyl 2‐(2‐methylenecyclopropyl) propyl ester	22	10.190	0.30
3‐Heptyne, 7‐fluoro‐2,2‐dimethyl‐	18	10.190	0.30
Butyl methyl isopropylphosphonate	14	10.190	0.30
Triacontyl acetate	16	10.298	0.43
Propanoic acid, 2,3‐dichloro‐, 1‐methylethyl ester	14	10.298	0.43
3‐Acetyloxypiperidin‐2‐one	12	10.298	0.43
3‐Thiophenecarboxylic acid	35	10.368	0.06
Cytosine	30	10.368	0.06
4(1H)‐Pyridinone, 2, 3‐dihydro‐1‐methyl	30	10.368	0.06
2‐Pyridinemethanol, 3‐hydroxy	46	10.427	0.21
2‐Isopropyl‐5‐methyl‐6‐oxabicyclo[3.1.0]hexane‐1‐carboxaldehyde	43	10.427	0.21
Methyl propyl ethylphosphonate	43	10.427	0.21
Cyclooctatin	38	10.531	0.96
(1R, 3R, 3aS, 4S, 7R, 9aR, 10aR, Z)‐7‐Isopropyl‐3, 4‐dimethoxy‐1‐(methoxymethyl)‐4, 9a‐dimethyl‐1, 2, 3, 3a, 4, 5, 7, 8, 9	27	10.531	0.96
Cis‐3‐methylpent‐3‐ene‐5‐ol	22	10.531	0.96
1‐(1,2‐Dimethoxypropyl)‐4‐methoxybenzene	78	10.646	0.09
Pyridine, 3‐butyl‐, 1‐oxide	72	10.646	0.09
Propanoic acid, 3‐bromo	32	10.797	1.47
Octanoic acid, 7‐methoxy‐3,7‐dimethyl‐, methyl ester	32	10.797	1.47
1,3‐Dioxolane, 2‐(5‐chloro‐3‐methyl‐3‐pentenyl)‐, (E)‐	27	10.797	1.47
3‐Methylbut‐2‐enoic acid, 2‐diethylaminoethyl ester	27	10.890	2.84
3‐Heptanone, 5‐methyl‐	14	10.890	2.84
2‐Imidazolidinone	14	10.890	2.84
(1R,5R)‐1,8‐Dimethyl‐4‐(propan‐2‐ylidene)spiro[4.5]dec‐7‐ene	95	10.955	0.13
(1R,5S)‐1,8‐Dimethyl‐4‐(propan‐2‐ylidene)spiro[4.5]dec‐7‐ene	93	10.955	0.13
Naphthalene, 1,2,3,5,6,8a‐hexahydro‐4,7‐dimethyl‐1‐(1‐methylethyl)‐, (1S‐cis)‐	95	10.984	0.15
Benzene, 1‐methyl‐4‐(1,2,2‐trimethylcyclopentyl)‐, (R)‐	95	10.984	0.15
Bicyclo[3.1.1]hept‐2‐ene, 2, 2′‐(1, 2‐ethanediyl)bis[6, 6‐dimethyl‐]	72	14.239	0.10
2,5‐Cyclooctadiene‐1‐carboxamide, N‐phenyl‐	50	14.239	0.10
beta.‐Ocimene	46	14.239	0.10
1,3,8‐p‐Menthatriene	45	14.405	0.19
6‐Isopropenyl‐3‐methoxymethoxy‐3‐methyl‐cyclohexene	38	14.405	0.19
Hexadecanoic acid, methyl ester	99	14.526	0.07
Pentadecanoic acid, 14‐methyl‐, methyl ester	95	14.526	0.07
Methyl 8,10‐octadecadiynoate	25	15.167	0.11
10,12‐Octadecadiynoic acid	17	15.167	0.11
Methyl 9,11‐octadecadiynoate	16	15.167	0.11
4‐Cyclohexylidene‐n‐butanol	30	15.371	0.06
Cyclohexene, 1‐methyl‐3‐(1‐methylethenyl)‐, (.+/−.)‐	30	15.371	0.06
Phenol, 3‐(dimethylamino)‐	25	15.371	0.06
Bicyclo[3.1.0]hexan‐2‐ol, 2‐methyl‐5‐(1‐methylethyl)‐, (1.alpha., 2.beta., 5.alpha.)‐	38	15.411	0.07
gamma.‐Terpinene	35	15.411	0.07
Cyclohexene, 1‐methyl‐4‐(1‐methylethylidene)‐	35	15.411	0.07
Icosa‐9,11‐diyne	42	15.658	0.22
o‐Mentha‐1(7), 8‐dien‐3‐ol	38	15.658	0.22
Glutaric acid, di (myrtenyl) ester	27	15.658	0.22
(1R,4r,7S)‐7‐Propylspiro[3.5]nonan‐1‐yl (4‐fluorophenyl)carbamate	47	15.381	0.07
cis‐3‐Hexenyl anthranilate	43	15.381	0.07
3‐Pyridineacetic acid	43	15.381	0.07
tert‐Butyl carbanilate	46	15.944	0.07
(1R,2S,4R)‐2,7,7‐Trimethylbicyclo[2.2.1]heptan‐2‐ol	38	15.944	0.07
Junicedranol	38	15.944	0.07
1,4,4,7a‐Tetramethyl‐2,4,5,6,7,7a‐hexahydro‐1H‐indene‐1,7‐diol	38	16.267	0.12
Benzo[f]chromen‐3‐one, perhydro‐2‐acetyl‐4a,7,7,10b‐tetramethyl	35	16.267	0.12
p‐Menth‐8‐ene, 3‐methylene‐	30	16.267	0.12
1,4,4,7a‐Tetramethyl‐2,4,5,6,7,7a‐hexahydro‐1H‐indene‐1,7‐diol	50	16.588	0.14
2(1H)‐Naphthalenone, 3, 4, 4a, 5, 6, 7‐hexahydro‐1, 1, 4a‐trimethyl‐	46	16.588	0.14
2,6,9,11‐Dodecatetraenal, 2,6,10‐trimethyl‐, (E,E,E)‐	49	16.770	0.19
1‐Adamantanecarboxylic acid chloride	38	16.770	0.19
Bicyclo[2.2.1]heptane, 7,7‐dimethyl‐2‐methylene‐	38	16.770	0.19
alpha.‐Farnesene	52	16.843	0.09
alpha.‐Pinene	30	16.843	0.09
2‐[1‐(Adamantan‐1‐ylamino)‐2,2,2‐trifluoro‐ethylidene]‐malononitrile	38	16.903	0.06
3,4‐Dihydroxybenzamide	30	16.903	0.06
N‐Hydroxy‐1,5‐dimethyl‐1H‐pyrrole‐2‐carboxamidine	30	16.903	0.06
Limonene oxide, trans	43	17.756	0.06
7‐Oxabicyclo[4.1.0]heptane, 1‐methyl‐4‐(1‐methylethenyl)‐	38	17.756	0.06
4,11‐Dimethyl‐8 (propan‐2‐yl) −5,12‐dioxatricyclo [9.1.0.04,6] dodecan‐7‐ol, Ac	35	17.756	0.06
Isobornyl isobutanoate	38	17.918	0.31
Imidazole, 4‐methyl‐5‐[2‐methyl‐2‐propenyl]‐	30	17.918	0.31
Bornyl acetate	27	17.918	0.31
Sesquithuriferol	62	18.772	0.08
1‐Naphthalenol, decahydro‐1,4a‐dimethyl‐7‐(1‐methylethylidene)‐, [1R‐(1.alpha.,4a.beta.,8a.alpha.)]‐	50	18.772	0.08
Cedranol,5‐neo‐	45	18.772	0.08

## 4 Discussion

The purpose of this study was to investigate the effects of MAO in a model of rheumatoid arthritis (RA) caused by complete Freund's adjuvant (CFA). To get a better understanding of how bone degradation and joint inflammation in RA are caused by the increased levels of proinflammatory cytokines such as TNF‐α, IL‐1β, IL‐6, NF‐κB, as well as decreased levels of IL‐4, we have targeted these mentioned cytokines in order to evaluate the role of MAO in the arthritic rat model. This study used the CFA‐induced arthritic rat model to establish the effects of MAO at three different doses (0.15 mL/kg, 0.3 mL/kg, 0.6 mL/kg). The CFA‐induced rheumatoid arthritis model was preferred because it closely resembles human arthritis with vascularization, cell division, inflammation, and synovial proliferation. Female rats were used because rheumatoid arthritis is more common and severe in women, and hormonal and immune factors likely contribute to this increased susceptibility.

Bone degeneration, bone erosion, and deformation are caused by cytokine triggering mechanisms, especially IL‐1 and TNF‐α (Ashiq et al. 2023; Saleem et al. 2023). Joint deformities, bone erosion, and decreased joint function can be linked to augmented levels of IL‐6, NF‐κB, and TNF‐α. Moreover, NF‐κB causes bone resorption, and prostaglandin‐producing COX‐2 is also thought to be involved in this study (Mobashar et al. 2020). The paw edema increased over time in all CFA‐treated groups due to the progressive nature of arthritis, but treatment with MAO significantly reduced paw swelling compared with the arthritic control group on days 14, 21, and 28, indicating attenuation of disease progression. MAO significantly reduces the overexpression of COX‐2 in RA. Furthermore, PGE2, another mediator that plays a crucial role in inflammation and joint damage by mediating COX‐2 (Mobashar et al. 2019; Parveen et al. 2023). A key limitation of our study was that, while docking results showed strong interaction between MAO compounds and COX‐2, we did not measure COX‐2 expression in this study. However, we did measure PGE2 levels, which showed a strong correlation and indirectly support the involvement of COX‐2.

High IL‐6 levels were associated with abnormalities in various hematological parameters, such as decreased red blood cells and hemoglobin and increased white blood cells and platelets. This finding supports the promise of MAO as an alternative treatment for RA, in line with current research on natural products with fewer side effects (Nazir et al. 2023). Furthermore, Li et al. highlighted the involvement of the ERK pathway and GRK2 deficiency in ECs, revealing the link between signaling pathways in arthritis development and treatment. The results of this study showed a decrease in inflammatory markers, arthritis scores, and paw edema. MAO demonstrates the ability to inhibit PGE2‐induced angiogenesis. In this study, MAO showed effects similar to piroxicam in easing joint swelling and balancing inflammatory markers. Piroxicam was selected as the reference drug because of its established anti‐inflammatory efficacy and widespread use in experimental arthritis models. Moreover, no mortality or visible signs of toxicity were observed in any treatment group. The absence of significant alterations in liver and kidney function biomarkers further suggests that the tested doses of *M. alternifolia* oil which are obtained from the previous literature were well tolerated during the study period. Although no major statistical differences were seen, the results suggest MAO could be a useful natural option for managing arthritis.

The GC–MS analysis of reported findings for phytocompounds of MAO revealed that these compounds significantly influenced AKT1, BCL‐2, COX‐2, MAPK3, and MMP9 targets, ultimately contributing to the development of rheumatoid arthritis. The stability of phytocompounds as possible drugs was highly supported by the favorable results of the linked SwissADME attributes of potential compounds for several models, including Lipinski rules, Lipinski violations, bioavailability score, and TPSA.

The process for identifying possible biological targets utilizing open databases was described in the network pharmacology analysis of rheumatoid arthritis. Out of the 5721 potential targets linked to rheumatoid arthritis, these databases found 247 potential targets for phytocompounds connected to the disease. A Venn diagram analysis identified 133 genes that matched, yielding 132 distinct targets that are related to rheumatoid arthritis and phytocompounds. Using these targets, a protein–protein interaction (PPI) network was built, exposing important hub nodes including AKT1, BCL‐2, COX‐2, MAPK3, MMP9, and many more. Network topology analysis based on degree centrality identified AKT1 as the major hub target, followed by BCL‐2, PTGS2 (COX‐2), MAPK3, and MMP9. These hub genes were closely associated with inflammatory and immune‐related pathways, including the TNF signaling pathway, Th17 cell differentiation, and Toll‐like receptor signaling pathway, highlighting their potential roles in the anti‐rheumatic effects of MAO. The PI3K/AKT/NF‐κB pathway, which includes AKT1, is involved in the regulation of inflammatory responses in RA. This pathway exacerbates the inflammatory phenotype in rheumatoid arthritis synovial fibroblasts (RASFs) by upregulating the expression of proinflammatory cytokines such as interleukin (IL)‐1α, IL‐1β, and tumor necrosis factor (TNF)‐α (Wang et al. 2018). B‐cell lymphoma 2 (BCL‐2) is an anti‐apoptotic protein that plays a significant role in the pathogenesis of rheumatoid arthritis (RA) by regulating the survival of various cell types within the synovial joint. BCL‐2 is highly expressed in RA synovial tissues, particularly in fibroblast‐like synoviocytes (FLSs), compared to osteoarthritis (OA) tissues. This overexpression contributes to the resistance of FLSs to apoptosis, leading to synovial hyperplasia and joint destruction characteristic of RA (Perlman et al. 2000). Cyclooxygenase‐2 (COX‐2) is an inducible enzyme that plays a pivotal role in the inflammatory processes associated with rheumatoid arthritis (RA). Upon stimulation by pro‐inflammatory cytokines, COX‐2 expression is upregulated in synovial tissues, leading to increased production of prostaglandins, which contribute to inflammation and pain in RA patients (Cai et al. 2024). Although COX‐2 expression was not directly measured, the reduction in PGE2 levels suggests that MAO may modulate this key inflammatory pathway. This supports the anti‐inflammatory effects observed in the treated groups. Moreover, Mitogen‐Activated Protein Kinase 3 (MAPK3), also known as extracellular signal‐regulated kinase 1 (ERK1), is part of the MAPK signaling pathway, that has been implicated in the regulation of pro‐inflammatory mRNA stability, affecting cytokines such as NF‐κB, TNF‐α and IL‐1β (Detka et al. 2024). Matrix metalloproteinase‐9 (MMP‐9) secreted by neutrophils and monocytes‐macrophages, actively participates in the degradation of extracellular matrix components, leading to cartilage and bone erosion in RA. This enzymatic activity facilitates the migration of pro‐inflammatory cells into the synovium, exacerbating the inflammatory response (Liu et al. 2024). GO enrichment analysis of these hub nodes examined a wide range of molecular functions (e.g., nuclear receptor activity, enzyme binding, nuclear steroid receptor activity, and protein kinase phosphatase activity), a number of biological processes (e.g., positive regulation of MAPK cascade, inflammatory response, positive regulation of cell population proliferation, and positive regulation of ERK1 and ERK2 cascade), and a number of cellular components (e.g., the external surface of the plasma membrane, neuronal cell body, cytoplasm, and extracellular regions). These targets are associated with KEGG pathways, which highlight important mechanisms (such as the TNF signaling pathway, Th17 cell differentiation, Toll‐like receptor signaling pathway, and others) that may have therapeutic relevance in the treatment of rheumatoid arthritis. These findings suggested that the phytocompounds analyzed may have significant potential in influencing these pathways and could be valuable in developing treatments for rheumatoid arthritis (Dennis et al. 2003; Thomas et al. 2007). Molecular docking further supported our results, indicating that there are moderate binding forces between core phytocompounds and biological targets. We built a model of “herb‐active compounds–targets–pathways” and found that citronellyl propionate, citronellyl formate, verbenone, isopiperitenone, linalool, and trans‐myroxide had a high relationship in the network, suggesting that they possess anti‐rheumatic properties. Molecular docking supported our findings by verifying the interaction between phytocompounds and their potential targets. Further research and clinical trials are required to examine the potential of MAO and validate its medicinal uses, even though we have provided some interesting results. The docking results indicate that MAO compounds have moderate binding affinities, and while MD simulations were not performed, future studies could provide further validation. Taken together, this study suggests that MAO may reduce RA symptoms by regulating cytokines, modifying the immune system, and protect bones, line with the present trend on safe and natural treatments. MAO exhibited a dose‐dependent anti‐arthritic effect, with the highest dose generally producing the most pronounced improvements in inflammatory, histopathological, and molecular parameters.

Higher levels of TNF‐α, IL‐1β, IL‐6, and PGE2 were associated with increased paw swelling and more severe histological damage, whereas IL‐4 showed a protective, inverse relationship. PGE2 levels also corresponded with NF‐κB activity, highlighting its role in inflammation. These observations underline the link between cytokine changes and arthritis severity in this model. Human protein structures were used for docking because rat structures were not available, and the high similarity between species supports the relevance of our results. Future studies could use rat‐specific models to confirm these findings.

GC–MS profiling indicated that the *M. alternifolia* oil exerts anti‐inflammatory effects owing to the presence of previously reported anti‐inflammatory and antirheumatic phytochemicals. Terpinen‐4‐ol exhibits anti‐arthritic activity primarily through the suppression of pro‐inflammatory cytokine expression (Aslam et al. 2022). Myrtenol has been shown to exert anti‐inflammatory and antinociceptive effects in experimental chronic arthritis, likely through direct modulation of neutrophil migration and enhancement of antioxidant activity (Gomes et al. 2017). Another study reported that synovial macrophages are recognized as promising therapeutic targets in osteoarthritis, and carveol stands out as a potential candidate for effective osteoarthritis management (Chen et al. 2024). D‐carvone prevents oxidative stress, inflammatory cell infiltration, and pathological alterations, thereby reducing the release of inflammatory cytokines and dampening the inflammatory response, ultimately protecting tissues and articular cartilage from damage (Chen et al. 2020).

Anethole produced marked anti‐inflammatory effects in the AIA group, reflected by the downregulation of cytokines and reduced nitric oxide levels (Ritter et al. 2017). The study demonstrated that combining trans‐anethole with methotrexate effectively inhibited arthritic progression in rats, with the added benefit of allowing a lower methotrexate dose, thereby reducing the adverse effects typically associated with high‐dose therapy (Bonetti et al. 2022). Linalool demonstrated dose‐dependent anti‐arthritic activity across multiple experimental models, underscoring its promise as a potential therapeutic agent for rheumatoid arthritis (Ahammed et al. 2025). Fenchone exerts strong anti‐inflammatory activity by inhibiting key pro‐inflammatory mediators, suggesting its therapeutic potential for chronic joint inflammation and other persistent inflammatory disorders (Nawaz et al. 2023). Gamma‐terpinene may serve as a natural alternative to NSAIDs and glucocorticoids, offering anti‐inflammatory benefits with fewer side effects (Silva et al. 2025). Bornyl acetate demonstrated antirheumatic activity in the CFA‐induced arthritis rat model (Neog et al. 2026). α‐Pinene's anti‐inflammatory and antioxidant properties indicate its potential therapeutic value in RA and in reducing the associated risk of atherosclerosis (Ali et al. 2026). Although GC–MS analysis identifies the phytochemicals present in the plant's oil, further research is needed to standardize the oil for therapeutic use. In the future, LC–MS analysis will also be required to detect a broader spectrum of phytochemicals.

This study focused on the pharmacological evaluation of whole *M. alternifolia* oil rather than dietary formulation; future work should address the need for future studies addressing formulation strategies, bioavailability enhancement, and dietary relevance. A limitation of this study is that mRNA was extracted from blood rather than synovial tissue, which would provide more localized insights into joint‐specific inflammation.

## 5 Conclusion

This study demonstrates that *M. alternifolia* oil (MAO) holds strong potential as a natural therapeutic agent for managing rheumatoid arthritis. In a CFA‐induced RA rat model, MAO effectively reduced paw swelling, arthritis scores, and joint tissue damage, while also helping to restore blood parameters. It significantly lowered pro‐inflammatory markers such as TNF‐α, IL‐1β, IL‐6, NF‐κB, and PGE2 and increased anti‐inflammatory cytokines like IL‐4, reflecting its immunomodulatory role. Network pharmacology highlighted several biological processes and pathways linked to RA, and molecular docking confirmed moderate binding of MAO with target sites. Altogether, the findings suggest that MAO may offer a multi‐targeted, well‐tolerated approach to RA treatment. It may also be proposed that the anti‐inflammatory and antirheumatic phytochemicals present in the plant's oil contribute to the ameliorative effects observed with *M. alternifolia* oil. Future research should focus on clarifying its molecular mechanisms and assessing its clinical potential in human studies.

## Author Contributions

**Sana Sabir:** writing – original draft, methodology. **Aisha Mobashar:** project administration, writing – original draft. **Hossam Kamli:** data curation, formal analysis. **Hafiza Mahnoor Baghdadi:** conceptualization, writing – original draft. **Bushra Akhtar:** writing – review and editing, methodology. **Amir Ali:** data curation, software. **Sana Saleem:** software, formal analysis. **Khalid Hussain:** writing – original draft, writing – review and editing. **Kiran Mashaal:** writing – original draft, writing – review and editing. **Esmael M. Alyami:** supervision, writing – review and editing. **Mona Alsolami:** investigation, validation. **Fakhreldeen Dabiellil:** writing – original draft, writing – review and editing. **Pravin Badhe:** resources, investigation. **Gehan M. Elossaily:** writing – review and editing, formal analysis. **Muhammad Shahab:** supervision, resources. **Abdel‐Rhman Z. Gaafar:** resources, supervision.

## Funding

This work is financially supported by the Ongoing Research Funding Program (ORF‐2026‐686), King Saud University, Riyadh, Saudi Arabia.

## Disclosure

ARRIVE Guidelines: The experimentation was conducted in accordance with applicable laws, and ARRIVE guidelines.

## Ethics Statement

The Institutional Research Ethics Committee of The University of Lahore, Lahore, Pakistan approved the study. Notably, all animal experimentation was conducted in accordance with applicable laws, regulations, and guidelines, prioritizing animal welfare and minimizing any potential harm. The responsible authorities approved water and *Phragmites australis* collections for research purposes. In addition, landowners also approved such collections for research purposes.

## Consent

The authors have nothing to report.

## Conflicts of Interest

The authors declare no conflicts of interest.

## Supporting information


**Figure S1:** Specifications of tea tree oil.

## Data Availability

Data will be available upon request from the corresponding author.
