# Supplementary material for: Unraveling the Anti‐Arthritic Potential of Bioactive Compounds From Melaleuca alternifolia Oil: Integrated In Vivo, Molecular Docking, and Network Pharmacology Insights
Source: Food Sci Nutr. 2026 Sep 23;14(9):e72328. doi: 10.1002/fsn3.72328 (PMC13598936; doi:10.1002/fsn3.72328)
Supplement: Supplementary file 1 [file FSN3-14-e72328-s001.docx]

**Supplementary Fig. 1:** Specifications of Tea Tree Oil
